# Co_1_/Ru Single‐Atom Alloy Catalyst for Sustainable Polypropylene Hydrogenolysis to Long‐Chain Liquid Products

**DOI:** 10.1002/adma.73643

**Published:** 2026-06-10

**Authors:** Yuzhen Ge, Yimeng Jin, Alexandra Krestnikova, Sibei Zou, Antonio J. Martín, Gonzalo Guillén‐Gosálbez, Javier Pérez‐Ramírez

**Affiliations:** ^1^ Institute of Chemical and Bioengineering Department of Chemistry and Applied Biosciences ETH Zurich Zurich Switzerland; ^2^ NCCR Catalysis Zurich Switzerland

**Keywords:** cobalt, polypropylene hydrogenolysis, ruthenium, single‐atom alloy catalyst, techno‐economic and life cycle analyses

## Abstract

Achieving selective polyolefin waste hydrogenolysis into fuels and chemicals requires architectures that disrupt Ru─Ru ensembles without compromising the intrinsic activity of the state‐of‐the‐art Ru catalyst. While metal modifiers can beneficially tune selectivity, they have been shown for high promoter‐metal contents, with associated penalties in structural definition and potential practicality. Here, we engineer cobalt speciation on Ru through impregnation to disclose how promotion spans the entire compositional space realizing a titania‐supported Co_1_/Ru single‐atom alloy catalyst (Co_10_Ru_90_, 3 mol% total metal content) that resolves these trade‐offs. Ex situ and operando analyses reveal that these bimetallic ensembles containing Ru─Co─Ru sites display a distinctively high barrier for over‐hydrogenolysis at late reaction stages while preserving the intrinsic activity of Ru. As a result, virgin polypropylene and post‐consumer waste were almost fully converted with a consistently high C_11+_ yield of up to 60% (78% total liquids), compared to <5% over the monometallic Ru counterpart. Process‐level simulations predict economically viable and environmentally sustainable operation enabled by the high single‐pass yields of long‐chain liquid products. These results reveal a wide compositional landscape for Ru promotion and recommend its second‐metal‐content single‐atom alloy limit as the most promising strategy for further catalyst design efforts.

## Graphical abstract

A titania‐supported Co_1_/Ru single‐atom alloy catalyst enables efficient polypropylene hydrogenolysis. Ru─Co─Ru motifs maintain Ru activity while suppressing excessive chain scission to gaseous products. Both virgin and waste plastics achieve high, environmentally and economically optimal C_11+_ liquid yields, supporting sustainable chemical recycling processes.

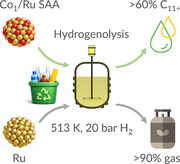

## 1 Introduction

As of 2024, global plastic production exceeded 450 million metric tons, with polyethylene (PE) and polypropylene (PP) accounting for 55%–60% [1, 2, 3]. Yet, only around 1% is chemically recycled on average and mainly via the well‐developed pyrolysis and gasification. These are energy‐intensive but highly effective routes for producing synthesis gas or short‐chain hydrocarbons with more challenging selectivity control toward longer products [4, 5, 6, 7, 8, 9, 10, 11]. Complementarily, hydrogenolysis of polyolefins into liquid alkane hydrocarbons under moderate conditions shows promise, but remains within laboratory walls mainly due to catalysts’ insufficient selectivity‐activity balance [12, 13, 14, 15].

State‐of‐the‐art supported Ru catalysts can efficiently activate polyolefin C─C bonds [16, 17, 18]. However, the strong affinity for reaction intermediates of contiguous Ru─Ru sites promotes over cleavage into low‐value gaseous and short‐chain liquid products [19, 20, 21] incompatible with economic and environmental feasibility [22]. Varying the geometric and electronic structure of Ru sites through suitable supports or metal modifiers can regulate intermediate adsorption and thus enhance selectivity toward longer‐chain products, though the extent is highly dependent on the nature of the polymer [23, 24, 25, 26, 27]. For example, alloying Ru with other metals (e.g., Mn, Fe, Co, and Ni) opens C─C cleavage pathways leading to less severe over‐hydrogenolysis [28, 29, 30]. In a previous work, our group demonstrated that titania‐supported Ru_1_Ni_3_ nanoparticles increased liquid product yields from 3% for monometallic Ru to ∼50% in consumer‐grade high‐density polyethylene (HDPE) hydrogenolysis, claiming favorable sustainability prospects. This performance was attributed to the in situ formation of Ru─Ni alloy domains that favor internal over terminal C─C cleavage through both hydrogenated and dehydrogenated intermediates [22]. Similar improvements in liquid product selectivity have recently been reported for CeO_2_‐supported Ru_1_M_3_ (M = Fe, Co, Ni) catalysts for low‐density polyethylene (LDPE) hydrogenolysis [31]. These gains in selectivity control, probably linked to disruption of contiguous Ru─Ru ensembles, are generally accompanied by reduced polymer conversion, while the precise nature of these alloys remains unclear.

This strategy of disrupting Ru─Ru ensembles with a second metal in materials with high M:Ru ratios has been recently taken to its extreme by a catalyst consisting on isolated ruthenium atoms (ca. 1.3 wt.%) in a matrix containing cobalt nanoparticles (ca. 49 wt.%) and aluminum (Co:Ru = 65:1 mol/mol) [32]. This catalyst offered ca. 80% yield to liquid products from LDPE, assigned to hydrogen spillover from Ru centers to adjacent Co ones in a single‐atom alloy structure. These results reveal that promotional effects may extend across a wide compositional range over the correct atomic structures and sparks the question of whether the symmetric strategy, i.e., using atomically‐dispersed low‐activity metals as selectivity modifier for ruthenium may achieve high performance over a well‐defined structure allowing mechanistic understanding while removing the need of high metal contents.

We impart speciation control through systematic compositional screening of the titania‐supported Ru─Co system for the hydrogenolysis of the under investigated polypropylene feedstock, identifying Ru─Co─Ru ensembles in a Co_1_/Ru single‐atom alloy (SAA) structure that retain the intrinsic activity of monometallic Ru while modulating late‐stage intermediate hydrogenolysis. This selective control strongly favors the formation of environmentally and economically optimal C_11+_ alkane [22] from the hydrogenolysis of post‐consumer goods. The SAA architecture enables a precise atomic description of sites [33, 34, 35, 36, 37], offering a strong platform to understand metal–metal interactions and derive mechanistic features able to tune selectivity in this complex reaction, while preserving the host metal's intrinsic activity.

## 2 Results and Discussion

### 2.1 Preparation and Structure‐Performance Relation of Supported Co*_x_* Ru*_y_* Catalysts

**FIGURE 1:**
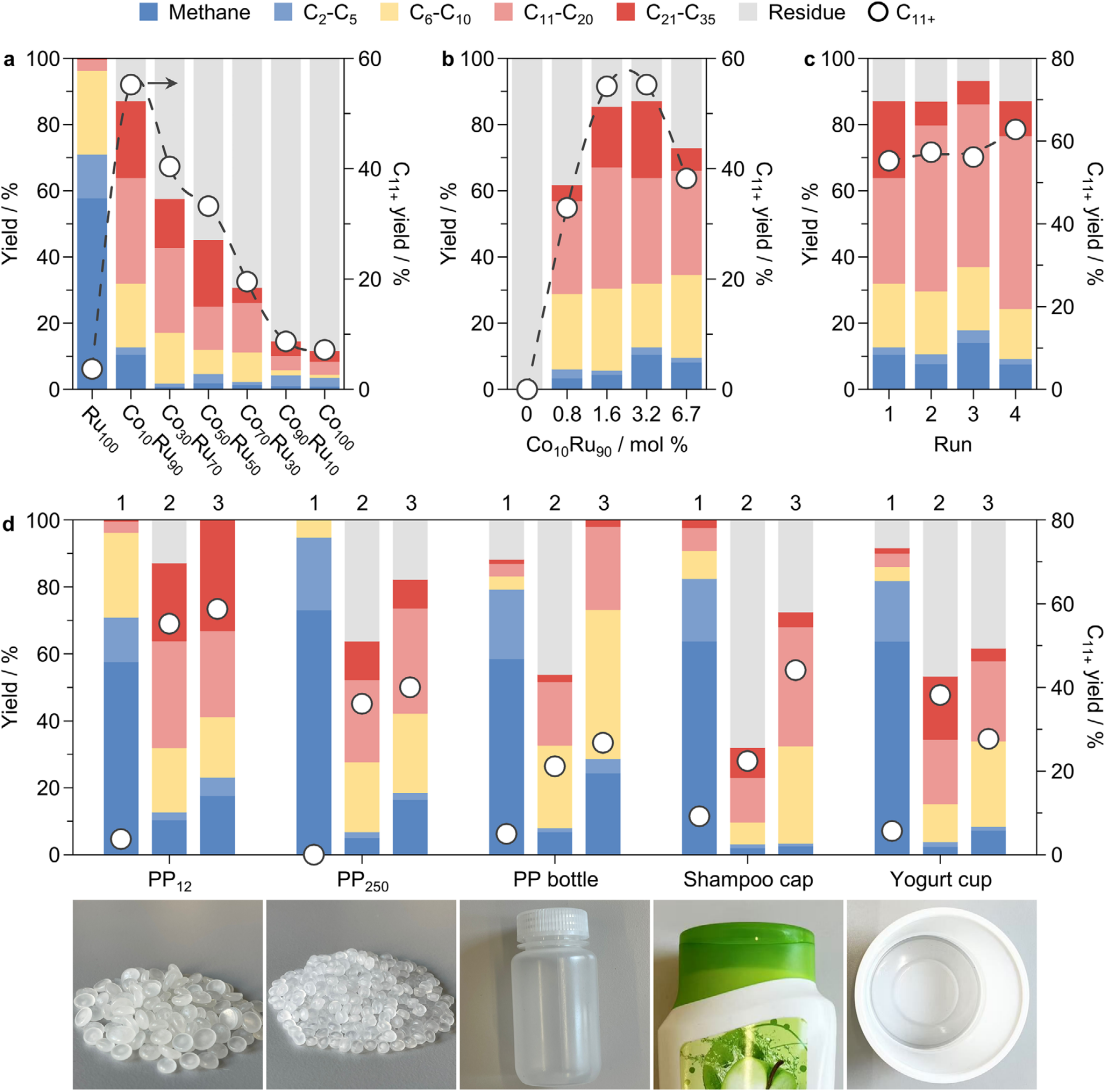
Catalytic performance for PP hydrogenolysis over TiO_2_‐supported Co*_x_* Ru*_y_* catalysts. Effect of (a) Co:Ru ratios and (b) total metal loading for Co_10_Ru_90_, keeping the total amount of Ru and Co used in each reaction. Detailed compositions are available in Table S1. (c) Reusability of Co_10_Ru_90_ over multiple reaction runs. (d) Performance obtained with different PP‐based feedstocks. 1, Ru_100_ after 12 h reaction. 2, Co_10_Ru_90_ after 12 h reaction. 3, Co_10_Ru_90_ after 24 h reaction. Reaction conditions (unless otherwise specified): 1.0 g PP_12_, 0.1 g catalyst, 513 K, 20 bar H_2_, 750 rpm, 12 h.

TiO_2_‐supported Co*_x_* Ru*_y_* catalysts were synthesized by wet impregnation, where *x* and *y* indicate the molar ratios of Co and Ru, respectively [18, 38]. Catalysts with a total metal loading of 3 mol% and varying Co:Ru ratios were firstly prepared (Table S1) and systematically evaluated under standard conditions: 0.1 g of catalyst, 1.0 g of PP_12_ (*M*_w_ = 12 kDa), 513 K, 20 bar H_2_, 12 h, 750 rpm. Monometallic Ru_100_ achieved complete PP_12_ conversion (defined as C_1_‐C_35_ yield) but predominantly produced gaseous products, including 58% methane and 13% C_2_‐C_5_ hydrocarbons, with only 4% C_11+_ long‐chain products (Figure 1a and Table S2). The incorporation of a small amount of Co in Co_10_Ru_90_ slightly reduced PP_12_ conversion to 87% but dramatically decreased gaseous product yield from 71% to 13% and simultaneously increased the C_11+_ yield to 55%, surpassing previously reported results (Table S3). Further increase of the Co content led to a pronounced decline in PP conversion until 12% for Co_100_, resulting in a volcano‐shaped dependence of C_11+_ yield on composition, with the maximum observed for Co_10_Ru_90_.

The catalytic behavior of Co_10_Ru_90_ samples with varying total metal content were then examined (Figure 1b and Table S2). Bare TiO_2_ showed negligible activity. Increasing metal loading first enhanced conversion, reaching 87% at 3.2 mol% then declining to 73% at 6.7 mol%. Long‐chain liquid products were in all cases the predominant fraction with the C_11+_ yield following a mild volcano trend, which could be attributed to geometric effects associated with nanoparticle size, influencing substrate adsorption and C─C scission efficiency [23, 24]. Notably, the Co_10_Ru_90_ catalyst with a total metal content of 1.6 wt.% (1.5 wt.% Ru and 0.1 wt.% Co) exhibited performance comparable to a previously reported Ru_1_/Co–LDH catalyst [32] (Table S4) exhibiting the inverse single‐atom alloy structure and developed for polyethylene processing, which contains a similar ruthenium content (1.3 wt.%) but a much higher cobalt one (49 wt.%). This result highlights the efficiency of the present catalyst design strategy leveraging low modifier contents.

The structural effect of the support was investigated by replacing TiO_2_ with various oxides (SiO_2_, Al_2_O_3_, ZrO_2_, and CeO_2_) while maintaining a constant Co_10_Ru_90_ metal loading (Table S5). The C_11+_ yield followed the order TiO_2_ > Al_2_O_3_ ≈ ZrO_2_ > CeO_2_ > SiO_2_. This trend can be attributed to differences in metal‐support interactions [39], with TiO_2_ promoting the formation of uniformly distributed Ru nanoparticles of optimal size, as evidenced by the XRD results (Figure S1), thereby enhancing catalytic performance. The apparent differences among Ru reflections strongly suggest that both too mild (SiO_2_) and too strong (CeO_2_) interactions result in suboptimal Ru nano‐structuring. Reusability tests of the optimal Co_10_Ru_90_ catalyst (3.2 mol%) showed stable performance over four cycles (Figure 1c and Table S6), with PP_12_ conversion at 87%–94% and C_11+_ yields of 55%–63%, highlighting its potential for practical application.

To evaluate the versatility of Co_10_Ru_90_ to valorize different feedstocks, control experiments with Ru_100_ as reference were tested for the conversion of PP_12_, PP_250_, and plastic waste (PP bottles, shampoo caps, and yogurt cups). Across all feedstocks, Ru_100_ produced predominantly gaseous products (>70%) with less than 10% C_11+_ hydrocarbons (Figure 1d and Table S7). In contrast, Co_10_Ru_90_ consistently suppressed gas formation below 13% and increased the C_11+_ product selectivity to above 50% under standard conditions (12 h). C_11+_ product yields varied across the series due to difference molecular weights and presence of additives interacting with the catalyst. Maximum values were 59% for PP_12_, 40% for PP_250_, 27% for PP bottles, 44% for shampoo caps, and 38% for yogurt cups.

**FIGURE 2:**
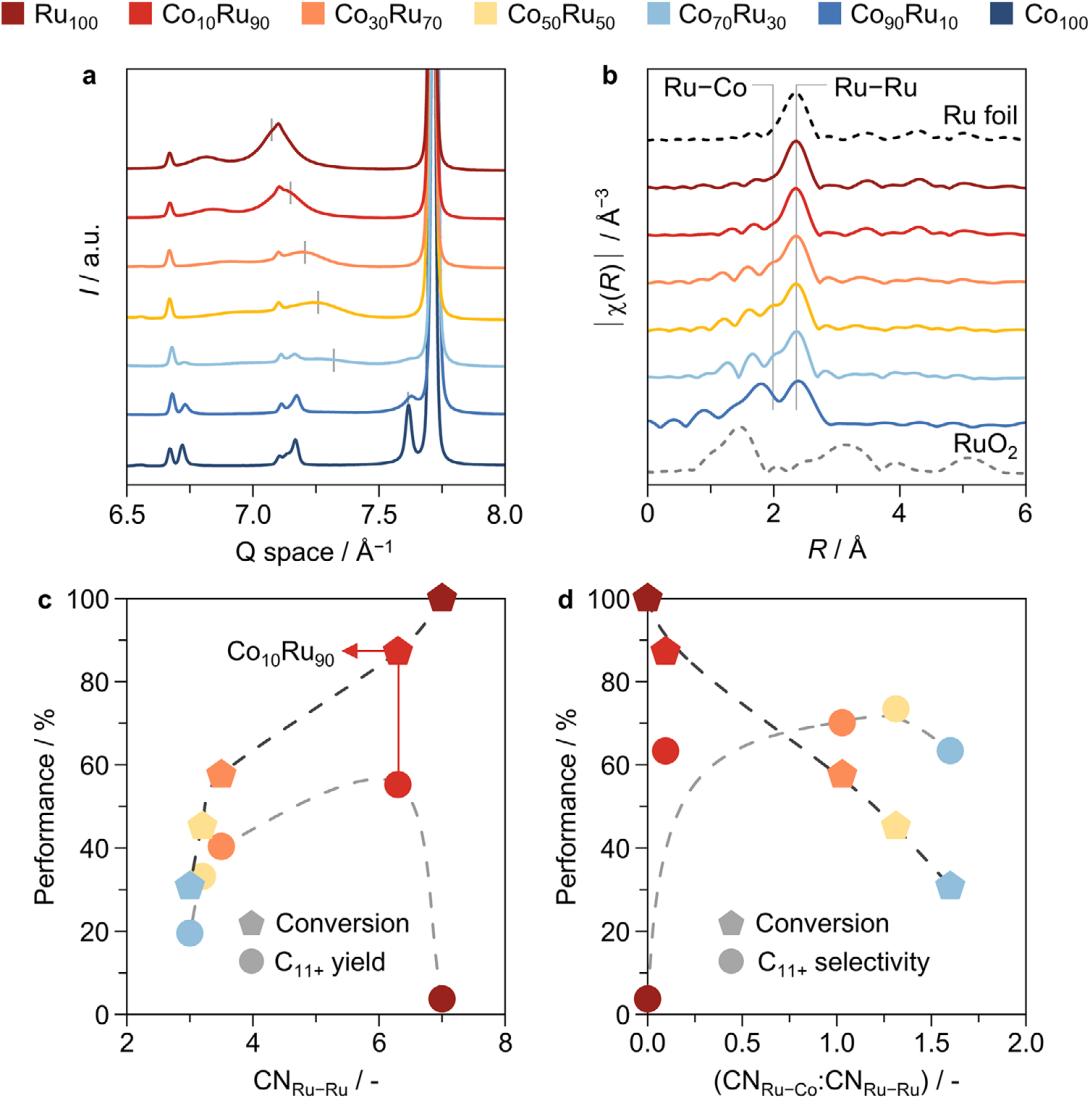
Structural characterization of TiO_2_‐supported Co*_x_* Ru*_y_* catalysts and their correlation with catalytic performance. (a) HRPD spectra. The gray lines indicate the characteristic diffraction peaks of CoRu species. (b) Ru K‐edge EXAFS profiles. C_11+_ yield and conversion (defined as C_1_‐C_35_ yield) of PP_12_ as a function of (c) CN_Ru–Ru_ and (d) CN_Ru–Co_:CN_Ru–Ru_ ratio. The color code for catalysts in the legend applies to all panels.

Catalyst characterization was carried out to preliminary elucidate the structure originating the enhanced performance resulting from Co incorporation into Ru. Synchrotron high‐resolution powder diffraction (HRPD), offering substantially higher resolution and sensitivity than conventional XRD, was employed to probe the crystallinity and for phase identification for the Co*_x_* Ru*_y_* catalysts (Figure 2a). Progressive shifts of the characteristic reflections from Ru_100_ to Co_100_ with increasing Co content preliminarily indicate Ru─Co interactions that were further explored by X‐ray absorption spectroscopy (XAS). Fourier‐transformed extended X‐ray absorption fine structure (EXAFS) analysis at the Ru K‐edge (Figure 2b and Figure S2), together with fitting results (Table S8), shows that the Ru─Ru coordination number (CN_Ru─Ru_) decreases from 7.0 in Ru_100_ to 3.0 in Co_70_Ru_30_ with increasing Co content, while the Ru─Co coordination number (CN_Ru─Co_) increases from 0.6 in Co_10_Ru_90_ to 4.8 in Co_70_Ru_30_. At higher Co contents (and thus lower Ru content), reduced spectral resolution precluded reliable fitting. These results early supported the formation of CoRu alloy nanoparticles. X‐ray absorption near‐edge structure (XANES) spectra at the Ru K‐edge (Figure S3) reveal a “white‐line” intensity (ca. 22117 eV) comparable to that of Ru metal foil and significantly lower than that of Ru oxide, indicating that Ru in the catalysts predominantly exists in the metallic state.

A correlation between catalytic performance and coordination number for Co*_x_* Ru*_y_* catalysts gave an initial insight into structure‐performance relationships. PP_12_ conversion increases monotonically with higher CN_Ru─Ru_, underscoring the role of contiguous Ru─Ru sites in promoting substrate conversion (Figure 2c). In contrast, the C_11+_ product yield exhibits a volcano‐shaped dependence on CN_Ru─Ru_, reaching a maximum at CN_Ru─Ru_ = 6.3 and CN_Ru─Co_ = 0.6 for Co_10_Ru_90_, indicating that introducing a small fraction of Ru─Co bonds is critical for achieving high selectivity toward long‐chain products. Further increases in CN_Ru─Co_ or CN_Ru─Co_:CN_Ru─Ru_ ratio did not improve C_11+_ selectivity and reduced PP_12_ conversion (Figure 2d and Figure S4). Based on these results, the Co_10_Ru_90_ catalyst was then examined in depth.

### 2.2 Identification of Co_1_/Ru SAA in Co_10_Ru_90_

**FIGURE 3:**
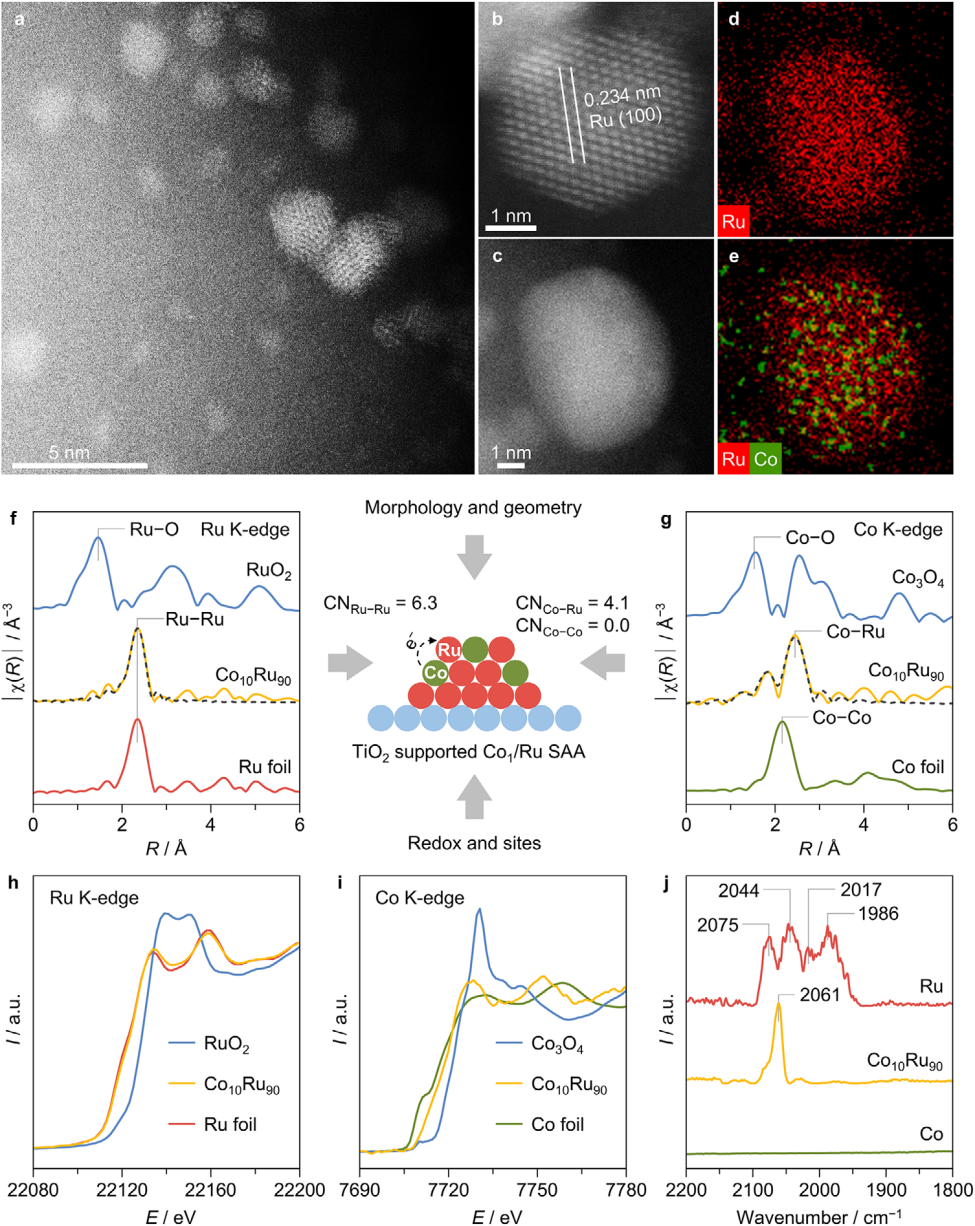
Structural characterization of TiO_2_‐supported Co_10_Ru_90_. (a–e) High‐resolution HAADF‐STEM images with corresponding EDX elemental mapping. EXAFS profiles and fitted curves (dashed lines) at the (f) Ru K‐edge and (g) Co K‐edge. XANES profiles at the (h) Ru K‐edge and (i) Co K‐edge. Corresponding metal foils and oxides were used as references for EXAFS and XANES analyses. (j) Steady state CO adsorption profiles for Ru_100_, Co_10_Ru_90_, and Co_100_ catalysts, with peak positions indicated.

Further characterizations were performed to identify the speciation governing Co_10_Ru_90_ catalysts. High‐angle annular dark‐field scanning transmission electron microscopy (HAADF‐STEM) reveals nanoparticles predominantly 2–5 nm in size, along with a minor population of smaller clusters (Figure 3a and Figure S5). Atomic‐resolution STEM images show a lattice spacing of 0.234 nm, corresponding to the (100) plane of hexagonal close‐packed Ru, indicating Ru‐rich nanoparticles (Figure 3b). Energy‐dispersive X‐ray spectroscopy (EDX) mapping of individual particles confirms a uniform Ru distribution, while Co appears as isolated angstrom‐scale signals (Figure 3c–e and Figure S6), further indicating the formation of a CoRu alloy.

The coordination environment of Co was studied through XAS analyses at the Co K‐edge (Figure S7) for freshly reduced ex‐situ samples. The Co─Ru coordination number (CN_Co─Ru_) and Co─Co coordination number (CN_Co─Co_) were 4.1 and zero in the optimal Co_10_Ru_90_ catalyst, respectively (Figure 3g). These results indicate that Co in Co_10_Ru_90_ interacts exclusively with Ru atoms, proving single‐atom Co alloyed within the Ru lattice (Ru─Co─Ru site, Co_1_/Ru SAA). Its XANES profile at Co K‐edges (Figure 3i and Figure S3) further indicate partial electron transfer from Co to Ru in Co_10_Ru_90_, evidenced by a shift of the Co K‐edge white‐line toward higher unoccupied states relative to metallic Co and closer to Co oxide [34]. Owing to the much higher Ru abundance (Figure 3f), the corresponding increase in Ru electron density is not clearly resolved at the Ru K‐edge (Figure 3h).

Although XAS confirms the Co_1_/Ru SAA structure of the Co_10_Ru_90_ catalyst, it does not provide information about the location of Co atoms. To further probe the existence of surface Co/Ru sites, steady‐state CO adsorption diffuse reflectance infrared Fourier transform spectroscopy (CO‐DRIFTS) was performed (Figure 3j and Figure S8). For Ru_100_, four CO adsorption bands of comparable intensity are observed: a low‐frequency band at 1986 cm^−1^ assigned to bridged CO on Ru─Ru sites, bands at 2017 and 2044 cm^−1^ corresponding to linear CO on contiguous Ru sites with high coordination [40, 41], and a higher‐frequency band at 2075 cm^−1^ attributed to linear CO adsorption at the Ru─TiO_2_ interface [42]. These features indicate the presence of diverse and continuous Ru surface sites. In contrast, Co_10_Ru_90_ exhibits a single CO adsorption band at 2061 cm^−1^, assigned to linear CO adsorption on Ru sites. The loss of multiple Ru─Ru‐related features indicates that Co incorporation disrupts Ru─Ru continuity, leading to a more uniform surface site distribution and the formation of Ru─Co─Ru ensembles. This observation is consistent with theoretical predictions of low surface segregation energies for CoRu alloys [43]. The upshift of the linear CO band in Co_10_Ru_90_ relative to Ru_100_ suggests weakened Ru→CO back‐donation due to isolation of Ru atoms by Co. Notably, no CO adsorption features associated with Co sites in Co_10_Ru_90_ are detected, consistent with the weak CO─Co interaction at room temperature and the absence of CO adsorption bands on Co_100_.

**FIGURE 4:**
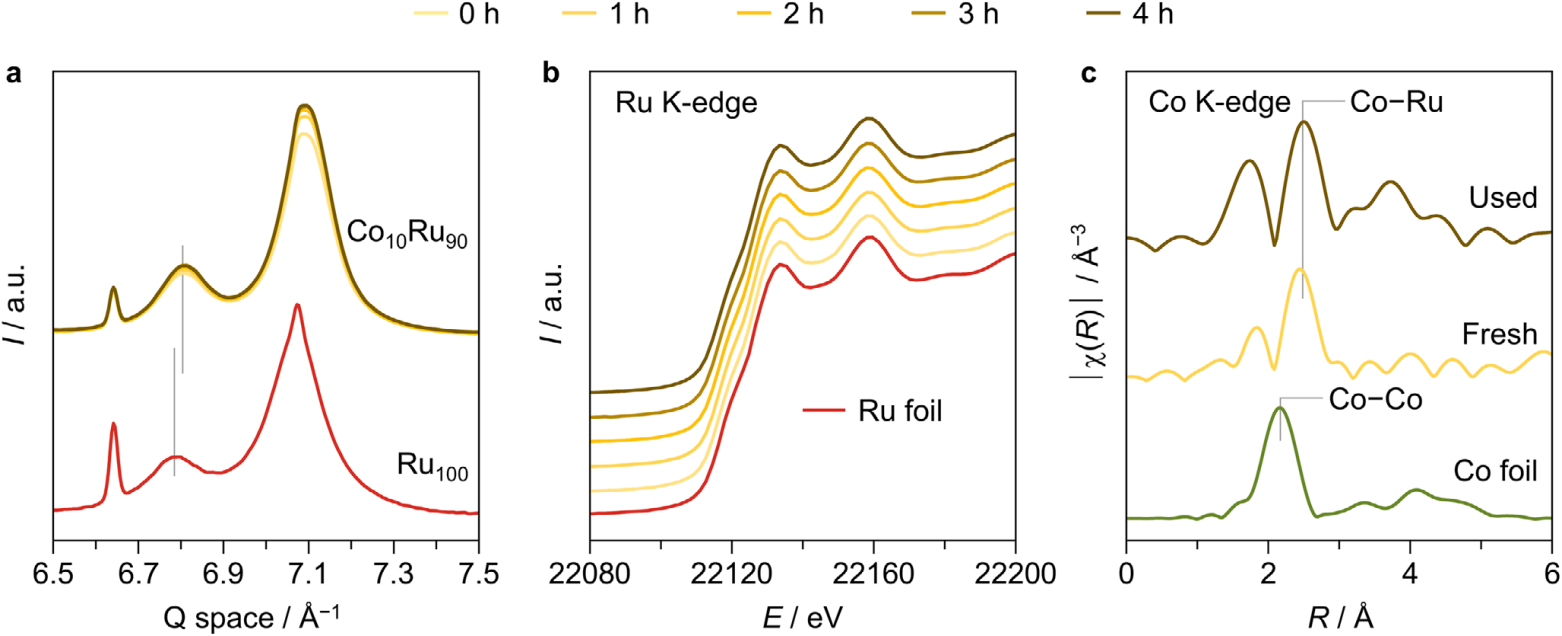
Time‐resolved operando characterization of the Co_1_/Ru SAA structure in TiO_2_‐supported Co_10_Ru_90_. (a) HRPD spectra, with TiO_2_‐supported Ru_100_ included as a reference; characteristic reflections emerging from Ru and CoRu nanoparticles are indicated. (b) XANES spectra at the Ru K‐edge. (c) EXAFS spectra at the Co K‐edge. Corresponding metal foils were used as references for both XANES and EXAFS analyses. Operando HRPD and XAS measurements were conducted synchronously under the following reaction conditions: PP_12_:catalyst = 1:1 mass ratio, 513 K, 20 bar H_2_, 4 h.

Operando XRD and XAS measurements confirmed the structural stability of the reference Co_10_Ru_90_, and Ru_100_ catalysts (Figures S9–S13). The XRD results indicate the formation of CoRu alloy phases compared to pure Ru, with stable reflections maintained throughout the measurement period (Figure 4a). XANES spectra at the Ru K‐edge for Co_10_Ru_90_ exhibit adsorption profiles similar to those of Ru foil, indicating the persistence of a stable metallic Ru phase during the entire measurement (Figure 4b). EXAFS spectra at the Co K‐edge show only Co─Ru coordination without detectable Co─Co contributions for both freshly reduced and spent catalysts from the operando experiment, confirming the stability of the Co_1_/Ru SAA structure under reaction conditions (Figure 4c).

For comparison, the set of TiO_2_‐supported Co_10_Ru_90_ catalysts with varying total metal loadings were also analyzed. HRPD patterns show consistent characteristic reflection positions across all samples (Figure S14), while decreasing peak intensities with lower metal loadings indicate similar Co─Ru interactions but reduced nanoparticle sizes. Consistent with this trend, XAS analysis and fitting (Figures S15–S17) confirm the preservation of the Co_1_/Ru SAA structure across all Co_10_Ru_90_ catalysts, with the Ru─Ru coordination number (CN_Ru─Ru_) decreasing from 7.1, 6.3, and 5.9 to 4.2 as the total metal loading is reduced from 6.7, 3.2, and 1.6 to 0.8 mol% (Table S9), respectively, indicating a progressive decrease in average particle size.

### 2.3 Preferential C─C Bond Scission Over Co_1_/Ru SAA

**FIGURE 5:**
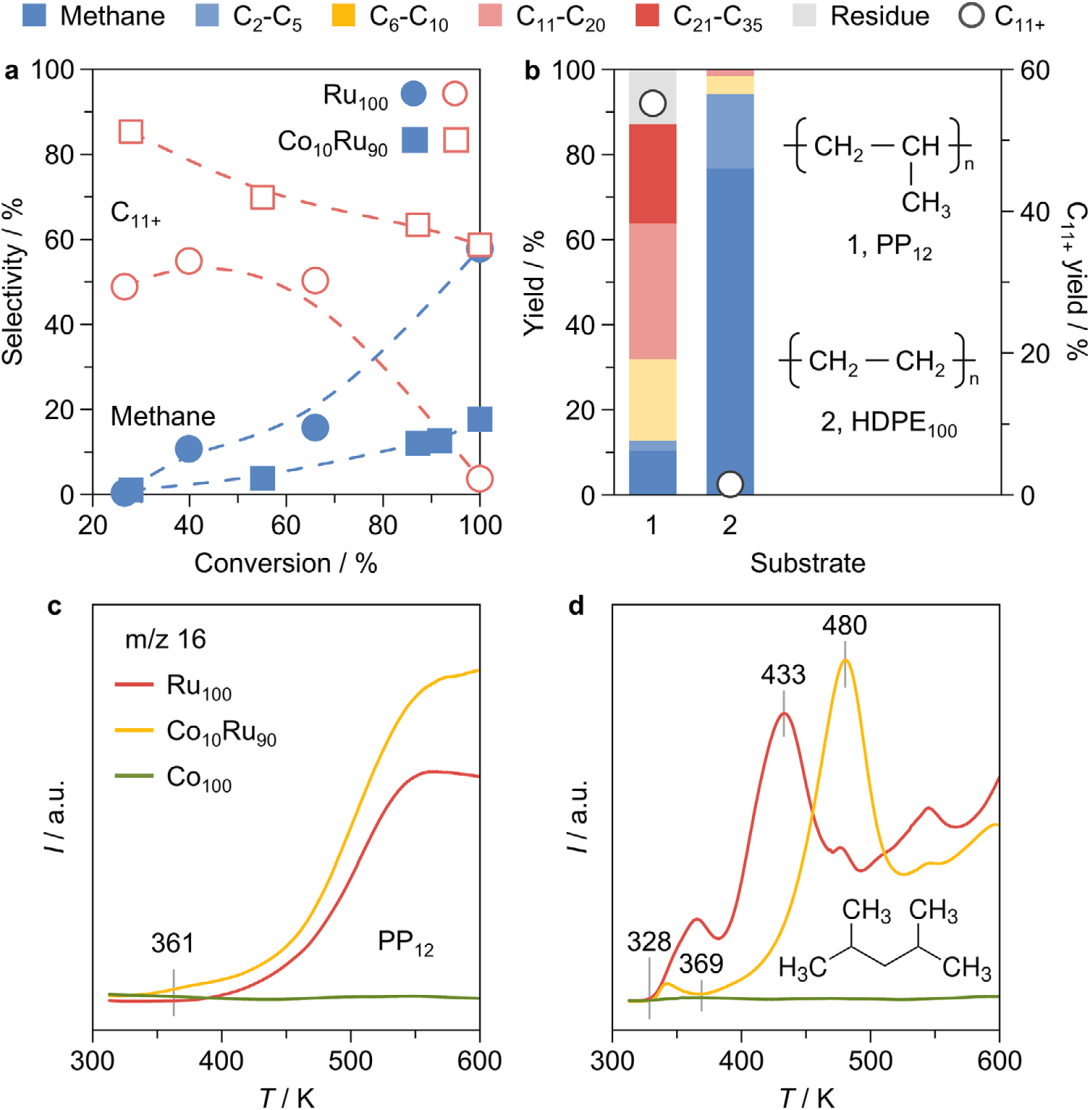
Control experiments elucidate catalyst and polymer‐dependent C─C bond scission behavior in hydrogenolysis reaction. (a) Selectivity to methane and C_11+_ products as a function of PP_12_ conversion. PP_12_ conversion was controlled by varying reaction time. (b) Catalytic performance of Co_10_Ru_90_ using (1) PP_12_ and (2) HDPE_100_ as feedstocks. Reaction conditions (unless otherwise specified): 1.0 g PP_12_, 0.1 g catalyst, 513 K, 20 bar H_2_, 750 rpm, 12 h. TPSR profiles of Ru_100_, Co_10_Ru_90_, and Co_100_ using (c) PP_12_ and (d) 2,4‐dimethylpentane as substrates. MS signal at *m/z* 16 corresponding to methane was monitored.

Further control experiments were conducted to elucidate the origin of the enhanced selectivity of Co_10_Ru_90_ catalysts toward long‐chain products. To probe intrinsic activity and selectivity, Ru_100_ and Co_10_Ru_90_ were evaluated at different PP_12_ conversions by varying reaction time (Figure 5a; Tables S10 and S11). At low PP_12_ conversion (<30%), selectivity toward gaseous products (C_1_−C_5_) remained minimal (<2%) for both catalysts, indicating that internal C─C bond cleavage within the polymer backbone initially dominates. As PP_12_ conversion increased to around 60%, gaseous product formation rose sharply for Ru_100_ but much more gradually for Co_10_Ru_90_. At this stage, C_11+_ selectivity remained around 50% for Ru_100_, while decreasing from 85% to 70% for Co_10_Ru_90_, reflecting concurrent formation and secondary cleavage of liquid intermediates. Importantly, Ru_100_ preferentially converted shorter liquids (C_6_−C_10_) into gases, whereas Co_10_Ru_90_ favored the conversion of long‐chain products (C_11+_) into shorter liquids. At high conversions (60%−100%), Ru_100_ showed pronounced over cleavage, with methane selectivity increasing from 16% to 58% and C_2_─C_5_ from 8% to 13%, accompanied by a drop in C_11+_ selectivity from 50% to 4%. In contrast, Co_10_Ru_90_ maintained high C_11+_ selectivity (59%) even at near‐complete conversion, indicating that moderated C─C scission and suppressed excessive hydrogenolysis extent to gaseous products.

Of note, both Ru_100_ and Co_10_Ru_90_ catalysts exhibited methane formation even at low PP_12_ conversion, indicating that methane generation occurs during the initial activation stage. To examine whether demethylation is intrinsic to PP activation, hydrogenolysis reactions were performed under identical conditions using PP_12_ (branched structure) and HDPE_100_ (*M*_w_ = 100 kDa, linear structure) over Co_10_Ru_90_ (Figure 5b and Table S12). PP_12_ reached 87% conversion with 11% methane and 13% total gaseous products yields, whereas HDPE_100_ was fully converted with markedly higher methane (77%) and total gas yields (94%) despite its longer average chain length. The substantially enhanced gas formation from HDPE_100_ indicates that internal C─C bond activation and scission along linear chains are rapid on Co_10_Ru_90_ catalyst. In contrast, for PP_12_, the presence of branched −CH_3_ groups might increase steric hindrance and weaken the interaction between catalyst and polymer. The removal of branched −CH_3_ groups likely constitutes the rate‐determining step for initial activation. Once demethylation occurs, subsequent main‐chain C─C cleavage may proceed readily.

Given the critical role of demethylation, temperature‐programmed surface reaction (TPSR) experiments were conducted, with methane evolution monitored by mass spectrometry (MS, *m/z* 16). As shown in Figure 5c, methane formation for both Ru_100_ and Co_10_Ru_90_ commenced at around 361 K and increased with temperature, whereas Co_100_ exhibited negligible methane production, indicating that Ru_100_ and Co_10_Ru_90_ possess comparable and substantially higher demethylation activity during the initial reaction stage. To probe the reactivity of liquid intermediates formed at later stages, 2,4‐dimethylpentane was employed as a model compound due to its structural similarity to PP_12_. In TPSR experiments with 2,4‐dimethylpentane (Figure 5d), methane formation over Ru_100_ initiated at 328 K and peaked at around 433 K, whereas for Co_10_Ru_90_ it began at a higher temperature (369 K) and peaked near 480 K. These results demonstrate that contiguous Ru─Ru sites in Ru_100_ promote more facile cleavage of intermediate liquid products into gaseous species, while Ru─Co─Ru ensembles in Co_10_Ru_90_ suppresse secondary over‐cleavage, consistent with its enhanced selectivity toward long‐chain hydrocarbons.

**FIGURE 6:**
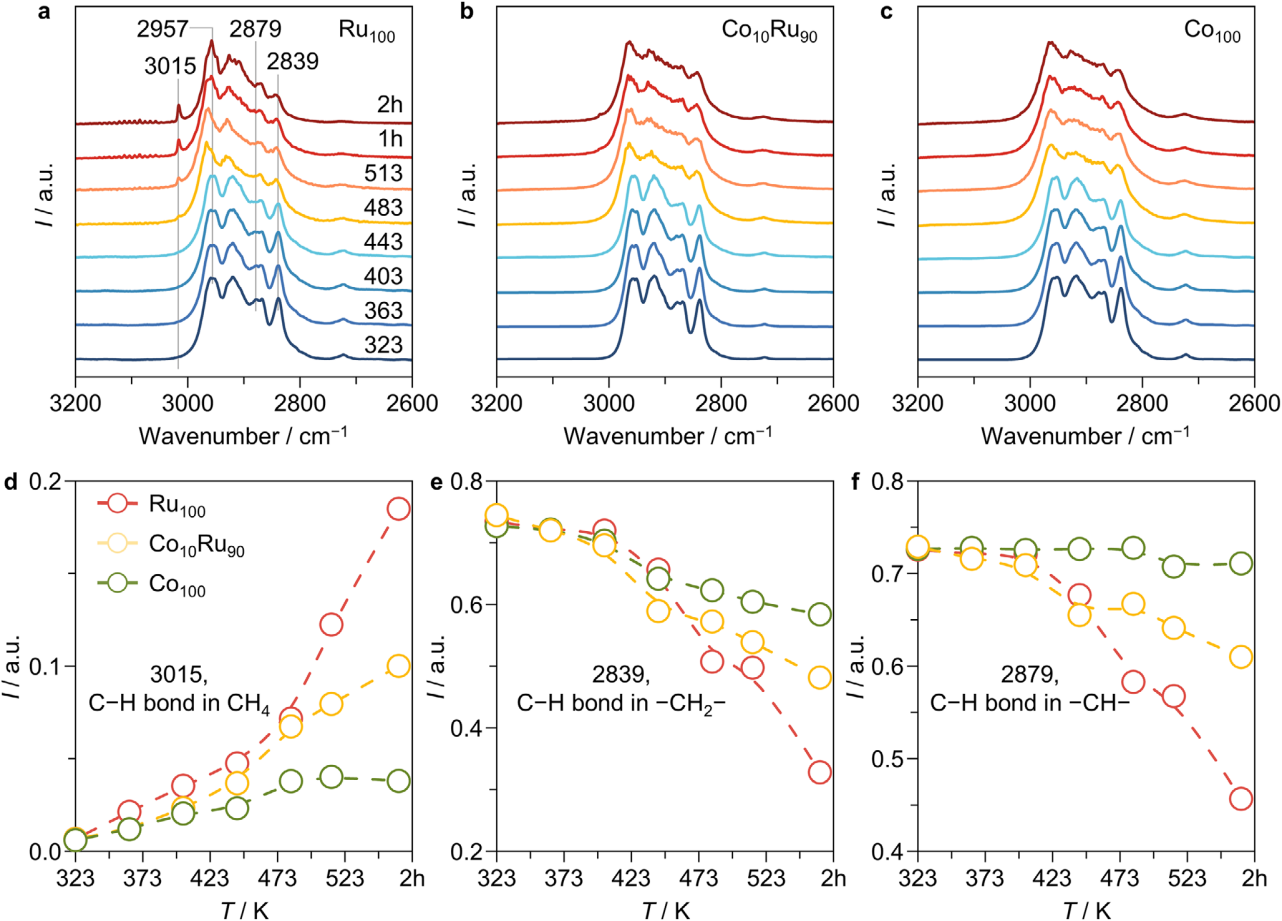
Temperature‐ and time‐resolved bond scission tracking in PP_12_ under reaction conditions. Operando DRIFTS spectra collected at varying temperatures and at 513 K over time for (a) Ru_100_, (b) Co_10_Ru_90_, and (c) Co_100_. (d–f) Corresponding evolution of peak intensities at indicated wavenumbers, attributed to C─H stretching vibrations in PP_12_. Reaction conditions: PP_12_:catalyst=1:1 mass ratio, 5 K min^−1^, 20 bar H_2_.

Operando DRIFTS was further employed to monitor the abundance of different C─H bonds during PP_12_ hydrogenolysis (Figure 6a–c). Bands at 3015, 2957, 2879, and 2839 cm^−1^ correspond to the asymmetric C−H stretch of methane, asymmetric −CH_3_, symmetric −CH−, and symmetric −CH_2_− vibrations of PP_12_, respectively [14]. Measurements were initiated at 323 K and increased stepwise by 20 K every 0.5 h up to 513 K, which was then held for 2 h. Time‐ and temperature‐resolved spectra (Figure 6d–f) show that methane intensity increased linearly with temperature up to 473 K for all catalysts, following the trend Ru_100_ > Co_10_Ru_90_ > Co_100_. Above 473 K, methane formation accelerated sharply on Ru_100_, increased moderately on Co_10_Ru_90_, and remained sluggish on Co_100_, in agreement with TPSR results. The appearance of methane at low temperatures indicates that branched −CH_3_ groups can be activated under mild conditions, although the reaction rate is strongly temperature dependent. Concurrently, the depletion of −CH_2_− and −CH− groups followed the same catalyst order, indicating that Ru_100_ promote more efficient C─C bond scission, while Co_10_Ru_90_ moderates excessive cleavage.

**FIGURE 7:**
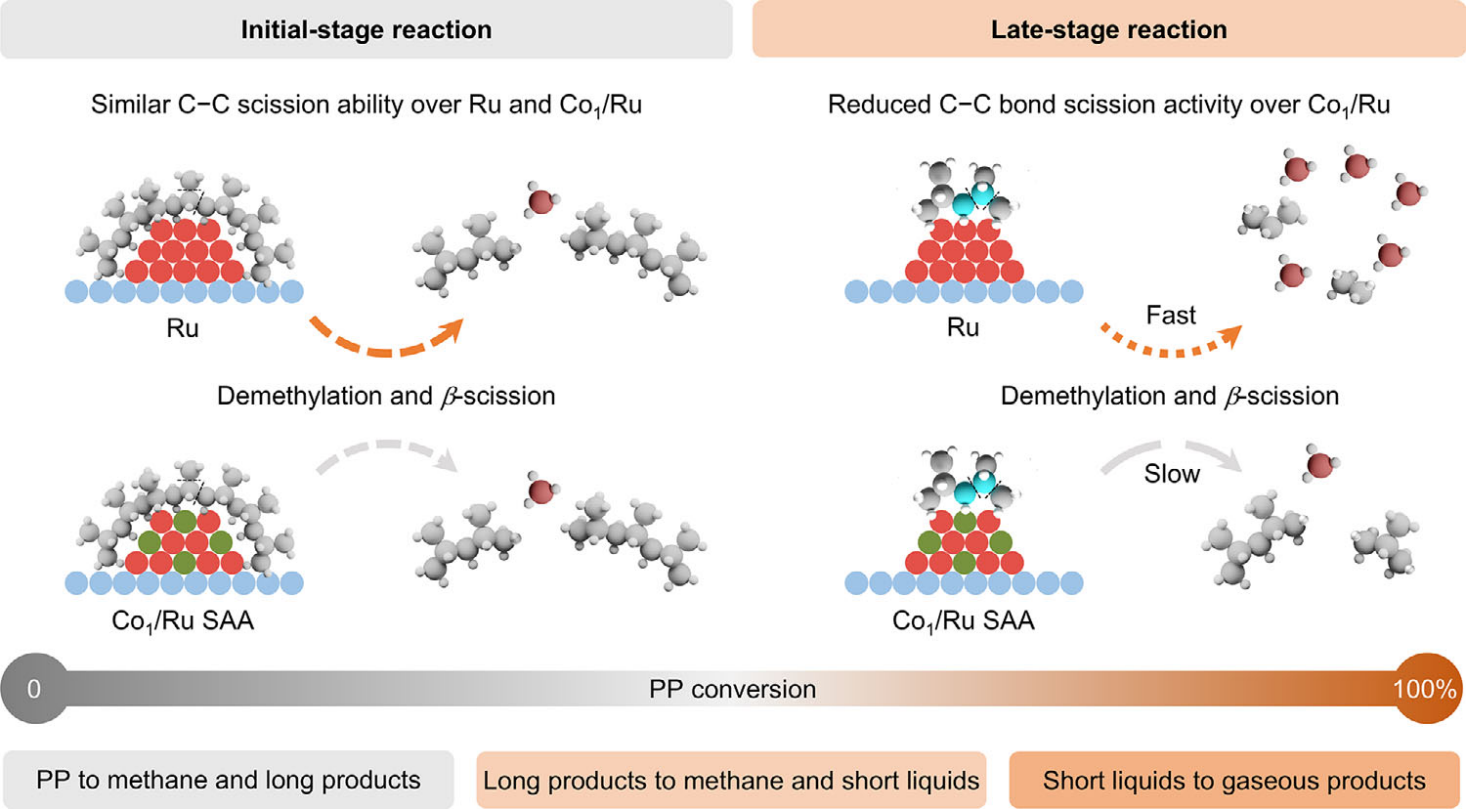
Proposed main mechanistic differences between Ru and Co_1_/Ru SAA for PP hydrogenolysis. The reaction proceeds in two conversion‐dependent stages: at low conversion, PP undergoes non‐selective demethylation and main‐chain *β*‐scission to form long‐chain intermediates on both Ru and Co_1_/Ru SAA catalysts; at high conversion, contiguous Ru─Ru sites promote over‐cleavage to gaseous products, whereas isolated Ru─Co─Ru ensembles in the Co_1_/Ru SAA suppress excessive C─C scission and preserve long‐chain liquid products.

To summarize the reaction mechanism, we propose a two‐stage pathway (Figure 7). In the initial stage, at low conversion, the large polymer chains adsorb or wrap on the catalyst surface and undergo demethylation of branched −CH_3_ groups to form methane, followed by main‐chain C─C bond scission (*β*‐scission) to generate long‐chain solid or liquid intermediates, with no pronounced difference between Ru and Co_1_/Ru SAA catalysts. As conversion increases, these intermediates are further processed into shorter liquid products. In the late stage, when long‐ and short‐chain liquid products dominate, catalyst‐dependent behavior emerges. On Ru, strong adsorption and C─C cleavage at contiguous Ru─Ru sites drive extensive over‐cleavage to gaseous products, limiting long‐chain liquid selectivity at high conversion. In contrast, isolated Ru─Co─Ru ensembles in the Co_1_/Ru SAA catalyst moderate scission, suppressing excessive cleavage and enabling sustained high yields of long‐chain liquid products under optimized conditions.

### 2.4 Techno‐Economic and Environmental Analyses

The pronounced selectivity differences between Ru_100_ and Co_10_Ru_90_ catalysts toward long‐chain liquid hydrocarbons motivated an environmental and economic assessment of a conceptual 20 t h^−1^ hydrogenolysis plant for PP waste conversion. Based on the process layout in Figure S18, the analysis includes catalyst regeneration and product separation units designed to reflect the experimentally observed product distributions. Scenarios using renewable hydrogen from wind‐ or solar‐powered electrolysis were compared with fossil‐derived hydrogen via steam methane reforming, while a business‐as‐usual, BAU case represents conventional fossil‐based hydrocarbon production and polypropylene life cycles.

**FIGURE 8:**
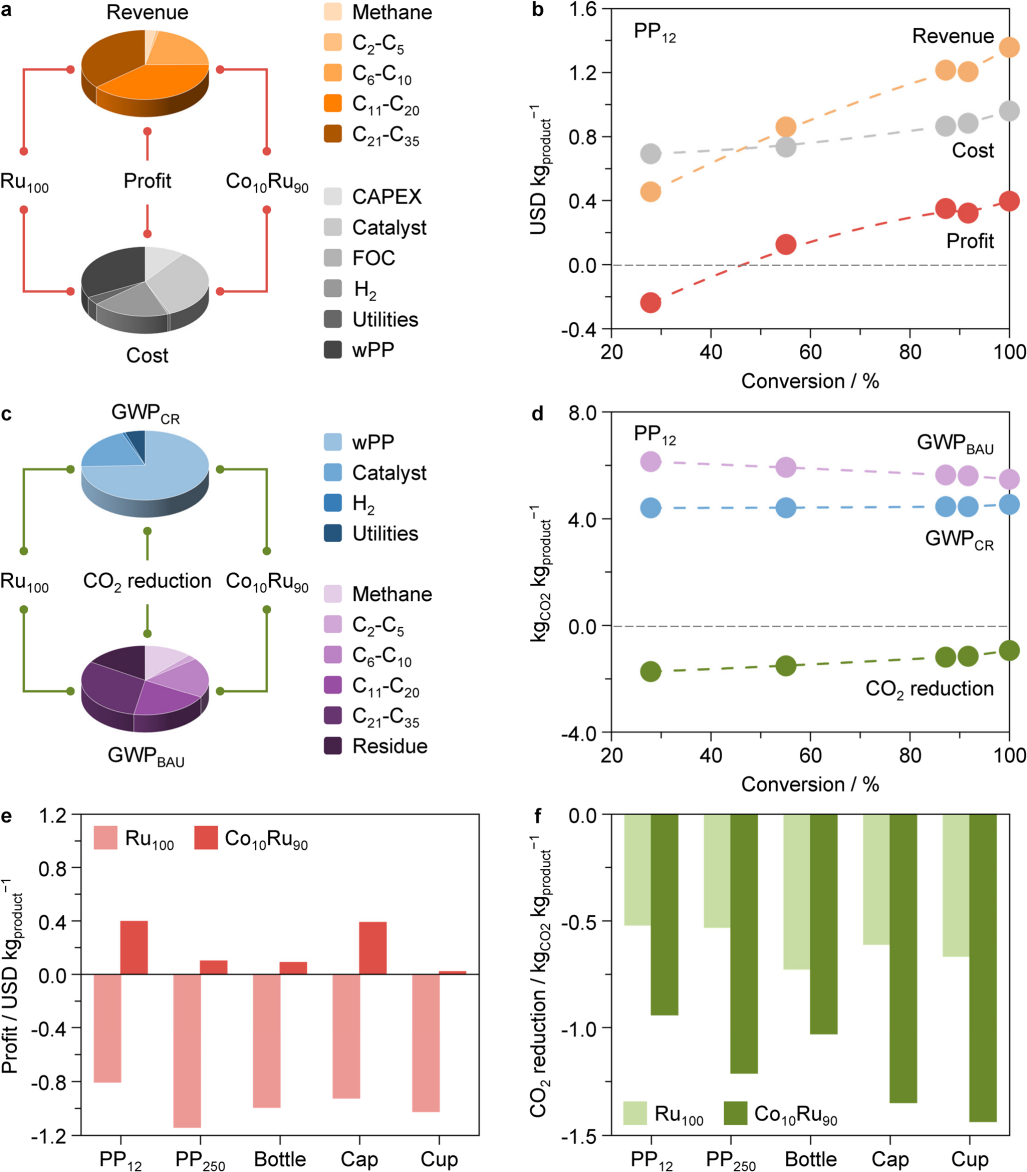
Techno‐economic and environmental analysis of large‐scale hydrogenolysis of PP items. (a) Example calculation of profit margin, defined as the difference between total product revenues at market prices and production costs. (b) Calculated revenues, costs, and profits at different conversions of PP_12_ using Co_10_Ru_90_ catalyst. (c) Example calculation of CO_2_ emissions reduction, defined as the difference between the global warming potential of the hydrogenolysis process (GWP_CR_) and that of the equivalent business‐as‐usual process (GWP_BAU_). (d) Calculated GWP_CR_, GWP_BAU_, and CO_2_ emissions reduction at different conversions of PP_12_ using Co_10_Ru_90_ catalyst. Calculated profits (e) and CO_2_ reduction (f) for different PP substrates using Ru_100_ and Co_10_Ru_90_ catalysts. Product yields were obtained from Figure 1d at a reaction time of 12 h for Ru_100_ and 24 h for Co_10_Ru_90_. Hydrogen produced through water splitting powered by wind energy was used in all analyses.

For the techno‐economic assessment (Figure 8a), revenues were estimated assuming all products were sold at market prices, while annual production costs included raw materials (H_2_, catalyst, and waste PP) and operational expenses associated with separation and recycling (Tables S13–S18). Profit was defined as the difference between total revenues and costs. The dependence of revenue, cost, and profit on PP_12_ conversion was evaluated using the optimal Co_10_Ru_90_ catalyst (Figure 8b and Table S19). At low conversion, costs exceeded revenues; however, as conversion increased, both rose until break‐even was reached at approximately 45%–50% conversion due to the high selectivity to long‐chain liquid products exhibited by Co_10_Ru_90_. Further increases in conversion led to steadily higher profits, reaching a maximum of 0.4 USD kg_product_^−1^. These results set a minimum conversion of ca. 50% under high selectivity to C_11+_ products to reach economic viability.

On the other hand, the environmental assessment indicates that producing liquid fuels via PP hydrogenolysis enables sequential utilization of fossil carbon: carbon atoms in polymers first serve as plastics and subsequently as fuel components. In contrast to the BAU scenario, which relies on separate fossil inputs for plastics and fuels, this circular pathway reduces overall fossil feedstock demand and lowers end‐of‐life emissions. By extending the lifetime of waste PP through hydrogenolysis, carbon release to the atmosphere is delayed, resulting in a decreased cradle‐to‐grave global warming potential (GWP_CR_, Figure 8c), where the entire life cycle is considered. The effect of PP_12_ conversion on reduction of CO_2_ emissions was further evaluated using Co_10_Ru_90_ (Figure 8d, Table S20). CO_2_ emissions were reduced across all conversions and increased only slightly at higher conversion, highlighting that economic and environmental performance for PP hydrogenolysis are only loosely coupled.

Similar techno‐economic and environmental analyses were applied to various PP feedstocks over Ru_100_ and Co_10_Ru_90_ (Figures S19 and S20) based on results presented in Figure 1d. Processes using Ru_100_ consistently yielded negative profits (Figure 8e and Tables S21), whereas Co_10_Ru_90_‐based systems were economically favorable due to higher product yields, improved product distributions, and lower hydrogen consumption. Concurrently, Co_10_Ru_90_ exhibited substantially lower GWP across all hydrogen supply scenarios (fossil, wind, and solar) compared with Ru_100_ (Figure 8f and Table S22), largely reflecting its reduced hydrogen and electricity requirements. Sensitivity analyses further reveal that Co_10_Ru_90_ maintains profitability and CO_2_ reductions under variable raw‐material and energy prices, market conditions, and life cycle uncertainties for PP caps (Tables S23 and S24), whereas performance is moderately feedstock‐dependent for bottles and cups (Figures S21 and S22). These results underscore that the Co_1_/Ru SAA design not only enables selective C_11+_ production but represents a solid starting point toward practical PP recycling.

## 3 Conclusions

In summary, this work shows that the promotional effects in Ru catalysts extend beyond modifier‐rich materials across a wide compositional range encompassing the opposite low‐content extreme up to the atomic dispersion. More broadly, it demonstrates how engineering of bimetallic catalysts can fundamentally reshape polypropylene hydrogenolysis, steering it toward economically and environmentally beneficial long‐chain liquid hydrocarbons. Multimodal structural and spectroscopic analyses identify isolated surface Co atoms embedded within Ru (Co_1_/Ru SAA) as the active architecture in TiO_2_‐supported Co_10_Ru_90_ catalysts. Operando and kinetic studies reveal that these Ru─Co─Ru ensembles moderate C─C bond scission and suppress late‐stage over‐cleavage compared to contiguous Ru─Ru sites, sustaining C_11+_ long‐chain product formation and extending performance to real‐world plastic waste feedstocks. Process modeling shows how atomic‐scale catalyst engineering translates directly into improved economic viability and reduced environmental impact. This low‐modifier single‐atom alloy limit overcomes key limitations of earlier architectures and emerges as the most advantageous design point for advancing both fundamental understanding and practical, selective, and sustainable polyolefin upcycling.

## 4 Experimental Section

### 4.1 Materials

Co*_x_* Ru*_y_* catalysts supported on TiO_2_ were prepared via a wet impregnation method, where *x*, *y* correspond to the molar percentage of Co and Ru, respectively. Typically, TiO_2_ (1.0 g, anatase, Sigma–Aldrich) was impregnated with calculated amounts of cobalt chloride precursor solution (40 mm CoCl_2_, Sigma–Aldrich) and ruthenium nitrosyl nitrate solution (Ru(NO)(NO_3_)*_a_* (OH)*_b_*, 0.015 g cm^−3^, *a *+ *b* = 3, Sigma–Aldrich). The resulting mixture was vigorously stirred at 333 K (200 rpm) until complete removal of the solvent. The obtained solid was vacuum‐dried at 343 K overnight and reduced at 673 K for 2 h under a flow of 50 vol% H_2_ in He (30 cm^3^ min^−1^). Catalysts with different compositions were synthesized by varying the precursor quantities. Co_90_Ru_10_ catalysts supported on SiO_2_, Al_2_O_3_, ZrO_2_, and CeO_2_ were synthesized using the same protocol. The Ru_1_Co‐LDH catalyst was prepared via a reported one‐step hydrothermal method [32].

### 4.2 Characterization

XRF measurements were conducted using a Rigaku ZSX Primus IV spectrometer. TPSR were performed using a Micromeritics Autochem II 2920 instrument equipped with a TCD and a mass spectrometer [44]. The catalyst (90 mg) was intimately mixed with PP_12k_ (10 mg, Sigma–Aldrich) and loaded into a U‐shaped quartz microreactor, followed by drying in He at 313 K for 1 h and heating in 5 vol% H_2_ in Ar (20 cm^3^ min^−1^) to 873 K at 5 K min^−1^ [45]. TPSR of the model molecule 2,4‐dimethylpentane were conducted following the same experimental setup and procedure. Prior to testing, the catalyst (100 mg) was impregnated with 2,4‐dimethylpentane and left in fume hood until it dried. HAADF‐STEM and EDX were acquired using either an aberration‐corrected JEM‐ARM300F microscope (GrandARM, JEOL) or a Talos F200X microscope (ThermoFisher Scientific) [45]. Synchrotron XAS and HRPD measurements were performed at the Swiss‐Norwegian beamlines (SNBL, BM31) of the European Synchrotron Radiation Facility (ESRF) [46]. Absolute energy calibration was conducted using metallic Ru and Co references. Both Ru and Co edges were continuously scanned with a 0.5 eV step size. HRPD patterns were collected using a Pilatus3 X CdTe 2M detector and averaged over 60 s [45, 47]. CO‐DRIFT were applied using a Bruker Invenio S FTIR [22]. The sample was first dried in He and then exposed to CO (10 vol% CO in He) for 1 h at room temperature to reach saturation coverage. The sample was then purged by He for 1 h to remove gas phase and physiosorbed CO. The spectra were continuously recorded throughout the entire procedure at 0.5‐min interval.

### 4.3 Operando Analyses

Operando DRIFT spectroscopy during PP_12_ hydrogenolysis was applied using a Bruker Invenio S FTIR [48] following this procedure: (i) the catalyst was mixed with PP_12_ in a 1:1 mass ratio and loaded in the cell, (ii) the sample was flushed with pure H_2_ and pressurized to 20 bar, (iii) background spectra were collected after stabilization of pressure and signal, and measurements were performed starting at 323 K, increasing by 20 K every 0.5 h until reaching 513 K, which was then maintained for 2 h; (iv) the system was naturally cooled down to room temperature. Spectra were recorded at 0.5‐min intervals with a resolution of 4 cm^−1^. Operando XAS and HRPD experiments were conducted under the same settings for ex‐situ samples at the same BM31 beamline. Catalyst and plastic were loaded between quartz wool plugs in a quartz capillary reactor (1.5 mm diameter, 0.01 mm thickness) [22]. The experiment procedure was as follows: (i) the freshly reduced catalyst was mixed with PP_12k_ in a 1:1 mass ratio and loaded in the cell; (ii) flushed with H_2_ and pressurized to 20 bar; (iii) data collection was initiated at room temperature, followed by heating to 513 K and stabilized for 4 h; (iv) the temperature was then decreased to RT. Consecutive XRD and XAS spectra at both the Ru K‐edge and Co K‐edge were acquired for the whole procedure.

### 4.4 Catalyst Evaluation

Catalytic performance was evaluated in a parallel pressurized batch reactor setup, as reported previously [19]. Typically, 1.0 g of PP and 0.1 g of catalyst were mixed in a glass inset and used for the reaction. Before the reaction, the reactor was purged sequentially with nitrogen and hydrogen before being pressurized with hydrogen to the desired pressure. The reaction mixture was then heated to the target temperature under mechanical stirring and held for the specified time. Upon completion, the reactor was cooled to ambient temperature by circulating chilled water.

### 4.5 Product Analysis

The products were analyzed following a previously reported method [22]. The glass inset was removed from the reactor and weighed to quantify the total gas produced. The composition of gaseous product was analyzed by GC (Agilent 6890 GC) equipped with an Agilent J&W PoraPlot Q column and FID. The liquid products and solid residue were dissolved by dichloromethane, sonicated and filtered using a syringe. The weight of tube, filter and syringe were measured before and after using in completely dry conditions, with the difference in mass corresponding to the amount of combined solid residue and catalyst. The total amount of liquid products was calculated by subtracting the gas product and solid residue from the amount of substrate used. The filtered liquid samples were analyzed in an Agilent 6890 GC equipped with an HP DB‐5 HT column to determining the distribution of products [19]. In the catalyst reusability test, the liquid and solid products retained in the insert were dissolved in cyclohexane instead of dichloromethane, with all other steps of the analytical procedure maintained unchanged.

### 4.6 Process Modeling

The PP hydrogenolysis was modeled in Aspen Plus v15 using the Peng‐Robinson thermodynamic package. The reaction was defined based on experimental results for conversion and selectivity over Ru_100_ and Co_10_Ru_90_ catalysts. The PP items were represented as a polyolefin model of consumer‐grade polymers, with a molecular weight of 250 kDa and an average waste PP feed rate of 20 t h^−1^. Additional details are provided in the Supporting Information.

## Author Contributions

Y.G. and Y.J. synthesized the catalysts, performed the catalytic evaluations and characterizations, and data analysis. Y.G. designed and performed operando experiments. A.K. conducted environmental and techno‐economic assessments under the supervision of G.G.‐G. S.Z. carried out electron microscopy investigations. A.J.M. coordinated the work. J.P.‐R. conceptualized and supervised the entire project and managed resources and funding. Y.G., A.J.M., and J.P.‐R. drafted and revised the manuscript. All authors approved the final version of the manuscript.

## Conflicts of Interest

The authors declare no conflicts of interest.

## Supporting information




**Supporting File**: adma73643‐sup‐0001‐SuppMat.pdf.

## Data Availability

Data presented in the main figures of the manuscript are publicly available through the Zenodo repository (http://doi.org/10.5281/zenodo.18368207). All other relevant source data are available from the corresponding author upon request. XAS data can be accessed at the ESRF database under the Experiment session: A31‐1‐307 on beamline BM31, with DOI number 10.15151/ESRF‐ES‐2312905716.

## References

[1] A. J. Martín , C. Mondelli , S. D. Jaydev , and J. Pérez‐Ramírez , “Catalytic Processing of Plastic Waste on the Rise,” Chem 7, no. 6 (2021): 1487–1533, 10.1016/j.chempr.2020.12.006.

[2] M. Q. Zhang , Y. Zhou , R. Cao , et al., “In‐Line NMR Guided Orthogonal Transformation of Real‐Life Plastics,” Nature 643, no. 8071 (2025): 395–403, 10.1038/s41586-025-09088-7. 40562941

[3] H. Li , H. A. Aguirre‐Villegas , R. D. Allen , et al., “Expanding Plastics Recycling Technologies: Chemical Aspects, Technology Status and Challenges,” Green Chemistry 24, no. 23 (2022): 8899–9002, 10.1039/d2gc02588d.

[4] K. Ragaert , L. Delva , and K. Van Geem , “Mechanical and Chemical Recycling of Solid Plastic Waste,” Waste Management 69 (2017): 24–58, 10.1016/j.wasman.2017.07.044. 28823699

[5] E. Selvam , Z. O. G. Schyns , J. A. Sun , et al., “Conversion of Compositionally Diverse Plastic Waste Over Earth‐Abundant Sulfides,” Journal of the American Chemical Society 147, no. 13 (2025): 11227–11238, 10.1021/jacs.4c18001. 40117193

[6] E. Selvam , K. Yu , J. Ngu , S. Najmi , and D. G. Vlachos , “Recycling Polyolefin Plastic Waste at Short Contact Times via Rapid Joule Heating,” Nature Communications 15, no. 1 (2024): 5662, 10.1038/s41467-024-50035-3. PMC11226686 38969641

[7] J. Ngu , S. Najmi , E. Selvam , et al., “Catalytic Deconstruction of Organic Additive‐Containing Plastics,” Nature Chemical Engineering 2, no. 3 (2025): 220–228, 10.1038/s44286-025-00187-w.

[8] J. Sun , J. Dong , L. Gao , et al., “Catalytic Upcycling of Polyolefins,” Chemical Reviews 124, no. 16 (2024): 9457–9579, 10.1021/acs.chemrev.3c00943. 39151127 PMC11363024

[9] S. Yang , Y. Li , M. Nie , et al., “Lifecycle Management for Sustainable Plastics: Recent Progress From Synthesis, Processing to Upcycling,” Advanced Materials 36, no. 33 (2024): 2404115, 10.1002/adma.202404115. 38869422

[10] S. Rejman , Z. M. Reverdy , Z. Bor , et al., “External Acidity as Performance Descriptor in Polyolefin Cracking Using Zeolite‐Based Materials,” Nature Communications 16, no. 1 (2025): 2980, 10.1038/s41467-025-57158-1. PMC11947190 40140345

[11] H. Li , J. Wu , Z. Jiang , et al., “Hydroformylation of Pyrolysis Oils to Aldehydes and Alcohols From Polyolefin Waste,” Science 381, no. 6658 (2023): 660–666, 10.1126/science.adh1853. 37561862

[12] L. Li , H. Luo , Z. Shao , et al., “Converting Plastic Wastes to Naphtha for Closing the Plastic Loop,” Journal of the American Chemical Society 145, no. 3 (2023): 1847–1854, 10.1021/jacs.2c11407. 36635072

[13] R. Gao , S. Mao , B. Lu , W. Liu , and Y. Wang , “Efficient Upcycling of Polyolefin Waste to Light Aromatics via Coupling C‐C Scission and Carbonylation,” Angewandte Chemie International Edition 64 (2025): 202424334, 10.1002/anie.202424334. 40104979

[14] X. Han , Y. Zhou , S. Chen , et al., “Hydrogen Spillover‐Induced Bronsted Acidity Enables Controllable Hydrocracking of Polyolefin Waste to Liquid Fuels,” Angewandte Chemie International Edition 64 (2025): 202505518, 10.1002/anie.202505518. 40296315

[15] A. Tennakoon , X. Wu , A. L. Paterson , et al., “Catalytic Upcycling of High‐Density Polyethylene via a Processive Mechanism,” Nature Catalysis 3, no. 11 (2020): 893–901, 10.1038/s41929-020-00519-4.

[16] S. D. Jaydev , A. J. Martín , M. E. Usteri , et al., “Consumer Grade Polyethylene Recycling via Hydrogenolysis on Ultrafine Supported Ruthenium Nanoparticles,” Angewandte Chemie International Edition 63, no. 11 (2024): 202317526, 10.1002/anie.202317526. 38105396

[17] L. Chen , L. C. Meyer , L. Kovarik , et al., “Disordered, Sub‐Nanometer Ru Structures on CeO_2_ are Highly Efficient and Selective Catalysts in Polymer Upcycling by Hydrogenolysis,” ACS Catalysis 12, no. 8 (2022): 4618–4627, 10.1021/acscatal.2c00684.

[18] P. A. Kots , S. Liu , B. C. Vance , et al., “Polypropylene Plastic Waste Conversion to Lubricants Over Ru/TiO_2_ Catalysts,” ACS Catalysis 11, no. 13 (2021): 8104–8115, 10.1021/acscatal.1c00874.

[19] S. D. Jaydev , A. J. Martín , D. Garcia , K. Chikri , and J. Pérez‐Ramírez , “Assessment of Transport Phenomena in Catalyst Effectiveness for Chemical Polyolefin Recycling,” Nature Chemical Engineering 1, no. 9 (2024): 565–575, 10.1038/s44286-024-00108-3. PMC11420077 39323546

[20] J. Qin , F. Wu , Y. Dou , et al., “Advanced Catalysts for the Chemical Recycling of Plastic Waste,” Advanced Materials 37, no. 23 (2025): 2418138, 10.1002/adma.202418138. 39748624

[21] M. Zhao , X. Chu , F. Wang , et al., “Enhancing the Conversion Efficiency of Polyethylene to Methane Through Codoping of Mn Atoms into Ru Centers and CeO_2_ Supports,” Journal of the American Chemical Society 146, no. 48 (2024): 33104–33111, 10.1021/jacs.4c10793. 39571077 PMC11669166

[22] I. Nogueroles‐Langa , Y. Ge , C. Salah , et al., “Polyethylene Hydrogenolysis to Liquid Products Over Bimetallic Catalysts With Favorable Environmental Footprint and Economics,” Nature Communications 16, no. 1 (2025): 9791, 10.1038/s41467-025-65260-7. PMC12603241 41213955

[23] J. Kim , S. Sun , D. Kim , et al., “The Role of Size and Structure of Catalytic Active Sites in Polyolefin Hydrogenolysis,” Chem Catalysis 4, no. 9 (2024): 101076, 10.1016/j.checat.2024.101076.

[24] J. A. Sun , P. A. Kots , Z. R. Hinton , et al., “Size and Structure Effects of Carbon‐Supported Ruthenium Nanoparticles on Waste Polypropylene Hydrogenolysis Activity, Selectivity, and Product Microstructure,” ACS Catalysis 14, no. 5 (2024): 3228–3240, 10.1021/acscatal.3c05927.

[25] M. Tamura , S. Miyaoka , Y. Nakaji , et al., “Structure‐Activity Relationship in Hydrogenolysis of Polyolefins Over Ru/Support Catalysts,” Applied Catalysis B: Environment and Energy 318 (2022): 121870, 10.1016/j.apcatb.2022.121870.

[26] M. Chu , X. Wang , X. Wang , et al., “Site‐Selective Polyolefin Hydrogenolysis on Atomic Ru for Methanation Suppression and Liquid Fuel Production,” Research 6 (2023): 0032, 10.34133/research.0032. 37040499 PMC10076030

[27] F. Liu , J. Zhou , X. Gao , et al., “Modulating Adsorption Behavior by Single‐Site Pt on RuO_2_ for Efficient Electrosynthesis of Glycolic Acid From Plastic Wastes,” Angewandte Chemie International Edition 64 (2025): 202422183, 10.1002/anie.202422183. 39985194

[28] C. Sun , J. Wang , J. Wang , et al., “Pt Enhanced C–H Bond Activation for Efficient and Low‐Methane‐Selectivity Hydrogenolysis of Polyethylene Over Alloyed RuPt/ZrO_2_ ,” Applied Catalysis B: Environment and Energy 353 (2024): 124046, 10.1016/j.apcatb.2024.124046.

[29] Q. Hu , S. Qian , Y. Wang , et al., “Polyethylene Hydrogenolysis by Dilute RuPt Alloy to Achieve H_2_‐Pressure‐Independent Low Methane Selectivity,” Nature Communications 15, no. 1 (2024): 10573, 10.1038/s41467-024-54786-x. PMC11618510 39632866

[30] X. Wang , R. Zhang , X. Wu , et al., “Enhancing Waste Plastic Hydrogenolysis on Ru/CeO_2_ Through Concurrent Incorporation of Fe Single Atoms and FeO_x_ Nanoclusters,” Angewandte Chemie International Edition 137, no. 27 (2025): 202506035, 10.1002/ange.202506035. 40289246

[31] Y. Yuan , Z. Xie , K. K. Turaczy , et al., “Controlling Product Distribution of Polyethylene Hydrogenolysis Using Bimetallic RuM_3_ (M = Fe, Co, Ni) Catalysts,” Chem & Bio Engineering 1, no. 1 (2024): 67–75, 10.1021/cbe.3c00007. PMC10906090 38434798

[32] M. Chu , W. Tu , Z. Zhuang , et al., “Efficient Polyolefin Upcycling Over Single‐Atom Alloy Catalyst,” CCS Chemistry 7 (2024): 2451–2464, 10.31635/ccschem.024.202404989.

[33] R. T. Hannagan , G. Giannakakis , M. Flytzani‐Stephanopoulos , and E. C. H. Sykes , “Single‐Atom Alloy Catalysis,” Chemical Reviews 120, no. 21 (2020): 12044–12088, 10.1021/acs.chemrev.0c00078. 32588624

[34] Y. Ge , X. Qin , A. Li , et al., “Maximizing the Synergistic Effect of CoNi Catalyst on Alpha‐MoC for Robust Hydrogen Production,” Journal of the American Chemical Society 143, no. 2 (2021): 628–633, 10.1021/jacs.0c11285. 33382262

[35] T. Zhang , A. G. Walsh , J. Yu , and P. Zhang , “Single‐Atom Alloy Catalysts: Structural Analysis, Electronic Properties and Catalytic Activities,” Chemical Society Review 50, no. 1 (2021): 569–588, 10.1039/d0cs00844c. 33170202

[36] C. He , Y. Gong , S. Li , et al., “Single‐Atom Alloys Materials for CO_2_ and CH_4_ Catalytic Conversion,” Advanced Materials 36, no. 16 (2024): 2311628, 10.1002/adma.202311628. 38181452

[37] G. Kyriakou , M. B. Boucher , A. D. Jewell , et al., “Isolated Metal Atom Geometries as a Strategy for Selective Heterogeneous Hydrogenations,” Science 335, no. 6073 (2012): 1209–1212, 10.1126/science.1215864. 22403387

[38] S. D. Jaydev , M.‐E. Usteri , A. J. Martín , and J. Pérez‐Ramírez , “Identifying Selective Catalysts in Polypropylene Hydrogenolysis by Decoupling Scission Pathways,” Chem Catalysis 3, no. 5 (2023): 100564, 10.1016/j.checat.2023.100564.

[39] W. Tu , M. Chu , X. Wang , et al., “SMSI‐Induced Charge Transfer for Selective Hydrogenolysis of Polyolefins,” Applied Catalysis B: Environment and Energy 339 (2023): 123122, 10.1016/j.apcatb.2023.123122.

[40] S. Y. Chin , C. T. Williams , and M. D. Amiridis , “FTIR Studies of CO Adsorption on Al_2_O_3_‐ and SiO_2_‐Supported Ru Catalysts,” Journal of Physical Chemistry B 110, no. 2 (2006): 871–882, 10.1021/jp053908q. 16471618

[41] Z. Wang , C. Dong , X. Tang , et al., “CO‐Tolerant RuNi/TiO_2_ Catalyst for the Storage and Purification of Crude Hydrogen,” Nature Communications 13, no. 1 (2022): 4404, 10.1038/s41467-022-32100-x. PMC9338308 35906219

[42] Y. Zhang , X. Yang , X. Yang , et al., “Tuning Reactivity of Fischer‐Tropsch Synthesis by Regulating TiO_x_ Overlayer Over Ru/TiO_2_ Nanocatalysts,” Nature Communications 11, no. 1 (2020): 3185, 10.1038/s41467-020-17044-4. PMC7314765 32581251

[43] A. V. Ruban , H. L. Skriver , and J. K. Nørskov , “Surface Segregation Energies in Transition‐Metal Alloys,” Physical Review B 59, no. 24 (1999): 15990–16000, 10.1103/PhysRevB.59.15990.

[44] Y. Ge , T. Zou , A. J. Martín , and J. Pérez‐Ramírez , “ZrO_2_‐Promoted Cu‐Co, Cu‐Fe and Co‐Fe Catalysts for Higher Alcohol Synthesis,” ACS Catalysis 13, no. 15 (2023): 9946–9959, 10.1021/acscatal.3c02534. 37560190 PMC10407844

[45] Y. Ge , A. J. Martín , M. Suvarna , et al., “Descriptors Toward Higher Alcohols and Olefins Formation in CO_2_ Hydrogenation on Promoted Iron Catalysts,” Applied Catalysis B: Environment and Energy 378 (2025): 125578, 10.1016/j.apcatb.2025.125578.

[46] W. van Beek , O. V. Safonova , G. Wiker , and H. Emerich , “SNBL, a Dedicated Beamline for Combined *In Situ* X‐Ray Diffraction, X‐Ray Absorption and Raman Scattering Experiments,” Phase Transitions 84, no. 8 (2011): 726–732, 10.1080/01411594.2010.549944.

[47] Y. Ge , T. Zou , A. J. Martín , et al., “Defective Zirconia Promotes Monometallic Iron Catalysts for Higher Alcohol Synthesis,” Chem Catalysis 4, no. 6 (2024): 101010, 10.1016/j.checat.2024.101010.

[48] M. Suvarna , T. Zou , S. H. Chong , et al., “Active Learning Streamlines Development of High Performance Catalysts for Higher Alcohol Synthesis,” Nature Communication 15, no. 1 (2024): 5844, 10.1038/s41467-024-50215-1. PMC11239856 38992019

