# Supplementary material for: Co1/Ru Single‐Atom Alloy Catalyst for Sustainable Polypropylene Hydrogenolysis to Long‐Chain Liquid Products
Source: Adv Mater. 2026 Jun 10;38(39):e73643. doi: 10.1002/adma.73643 (PMC13361257; doi:10.1002/adma.73643)
Supplement: Supplementary file 1 — Supporting File: adma73643‐sup‐0001‐SuppMat.pdf. [file ADMA-38-e73643-s001.pdf]

Supporting Information

Co_1/Ru single atom alloy catalyst for sustainable polypropylene hydrogenolysis to long-chain liquid products

Yuzhen Ge,[1,2] Yimeng Jin,[1] Alexandra Krestnikova,[1,2] Sibei Zou,[1,2] Antonio J. Martín,[1,2] Gonzalo Guillén-Gosálbez,[1,2] and Javier Pérez-Ramírez[1,2]*

[1] Institute of Chemical and Bioengineering, Department of Chemistry and Applied Biosciences, ETH Zurich, Vladimir-Prelog-Weg 1, 8093 Zurich, Switzerland.

[2] NCCR Catalysis, 8093 Zurich, Switzerland.

* Corresponding author. E-mail: jpr@chem.ethz.ch

Table of Contents

Supporting Methods..2

Supporting Tables..6

Supporting Figures..30

Supporting References..52

Supporting Methods

Process modeling of PP hydrogenolysis

The PP hydrogenolysis was modeled in Aspen Plus v15 using the PENG-ROB property method, applying the Peng-Robinson cubic equation of state, which was chosen for its suitability for non- or mildly polar mixtures containing hydrocarbons and light gases.[1] The reaction was defined based on experimental results for conversion and selectivity over Ru_{100} and $Co_{10}Ru_{90}$ catalysts and the PP items were modeled as a polyolefin mimicking consumer-grade polymers with a molecular weight of 250 kDa and an average input flow of waste PP of 20 t h^{-1}, following assumptions by Salah et al.[2]

The process flowsheet is shown in **Figure S18** and includes the hydrogenation step, a catalyst separation and recycling step and a cascade of product separation columns. PP (25 °C, 1.013 bar), and hydrogen (40 °C, 20 bar), are fed to the process in stoichiometric proportions. The feed streams are first mixed (M1) and subsequently heated to the reaction temperature (250 °C) in the heat exchanger (H1). The reaction takes place in the reactor (R1), modeled as an RStoic reactor block, operating at 250 °C and 20 bar. The catalyst enables the conversion and selectivity specified in **Table S7** under identical conditions. The reactor effluent is cooled to 40 °C (C1) and depressurized to 1.013 bar (V1), then directed through a catalyst regeneration filter in the presence of n-hexane. The regenerated catalyst is recycled back to the reactor (R1), while the resulting mixture, comprising the reaction products, solvent, and unreacted materials proceeds to the downstream processing section. To recover the liquid fraction, the filtered stream undergoes a flash separation step, where the liquid and vapor streams are recompressed to 25 bar using a pump (P1) and a compressor (K1), respectively. Excess hydrogen in the recompressed vapor stream is separated and recovered via a pressure swing adsorption (PSA) unit. The remaining streams are mixed (M3) and cooled to 40 °C (C2).

Methane, light gases, gasoline, diesel, and motor oil, which constitute the five hydrogenolysis products, are separated to high molar purity in six distillation columns (D1-D6), modeled with the DSTWU column model. The *n*-hexane solvent used for catalyst regeneration is recovered in the top fraction of column D4, combined with the fresh *n*-hexane in the mixer (M2), and then fed back to the catalyst filter. A make-up stream of *n*-hexane is required to compensate for process losses, primarily associated with product recovery. Finally, heat integration was conducted using Aspen Energy Analyzer v15, providing the design specifications for utility streams and heat exchanger areas.

Techno-economic and life cycle assessment

Techno-economic and environmental assessments were performed, based on the process described above, to study the economic and environmental impacts of PP hydrogenolysis using Ru_{100} and $Co_{10}Ru_{90}$ catalysts. The mass and energy balances resulting from the simulation were used to build the life cycle inventories (LCI) and calculate the costs and revenues.

For the techno-economic assessment (TEA), the total annualized cost (TAC) was calculated based on the cost factors and correlations defined by Sinnott and Towler[7], as shown in equation (1).

$$TAC = VOC + FOC + ACC \quad (1)$$

The TAC considers variable operating cost (VOC), including costs of raw materials, electricity, utilities and waste treatment operations, as well as the fixed operating cost (FOC) and annualized capital cost (ACC). The FOC and ACC both depend on the capital expenditures (CAPEX), which were calculated using the cost correlations from Sinnott and Towler.[3]

The CAPEX corresponds to the purchased equipment cost (C) adjusted by installation factors, where the purchased equipment cost is based on a sizing factor (S) and parameters a, b and n, following equation (2). The parameters (a, b, and n) are unique to each type of equipment and the values used to calculate the equipment cost can be found in **Table S13**.

$$C = a + bS^n \quad (2)$$

The purchased equipment cost is then used to determine the inside battery limit cost (ISBL), applying typical installation factors for "fluids-solids" processes for all plant equipment units (j = 1, ..., M), as described by Sinnott and Towler and shown in equation (3)[7].

$$ISBL=\sum_{j=1}^{M} C_j \left[(1 + f_p)\, f_{m,j} + (f_{er} + f_{el} + f_i + f_c + f_s + f_l)\right] \quad (3)$$

The installation factors consider corrections for piping (f_p = 0.6), equipment material (f_m = 1 for carbon steel and 1.3 for 304 stainless steel), equipment erection (f_{er} = 0.5), electrical installation (f_{el} = 0.2), instrumentation and control (f_i = 0.3), civil engineering services (f_c = 0.3), structure and building services (f_s = 0.2), and lagging and paint services (f_l = 0.1). For this process we assume that, except for the reactor and the PSA unit, which involve hydrogen and hence require 304 stainless steel, all equipment is made of carbon steel.

To find the fixed capital cost (FCC), the ISBL need to be adjusted to account for offsites (OS), design and engineering (D&E) and contingency (X) as shown in equation (4) below.[3]

$$\mathrm{FCC} = \mathrm{ISBL}\,(1 + \mathrm{OS})\,(1 + \mathrm{D\&E} + \mathrm{X}) \tag{4}$$

To annualize this fixed capital investment, the annual capital charge ratio (ACCR) is required, which corresponds to the fraction that must be paid out each year to fully repay the capital investment cost, taking into account principal and accumulated interest (i) over the plant lifetime (n), as given by equation (5). The ACCR was calculated assuming a plant lifetime of 25 years and an interest rate of 10%, according to the variables described in **Table S14.**

$$\mathrm{ACCR} = \frac{i\,(1 + i)^n}{(1 + i)^n - 1} \tag{5}$$

Finally, the ACC was calculated from the resulting FCC and the annual capital charge ratio (ACCR), as shown in equation (6) below.

$$\mathrm{ACC} = \mathrm{FCC} \cdot \mathrm{ACCR} \tag{6}$$

Using the ISBL, FCC and cost of labor (CL), the FOC can be assessed, according to the framework described by Sinnott and Towler[7]. It is determined following equation (7) and includes costs associated with operating labor (CL), supervision (f_{sup} = 0.25), salary overheads (f_{so} = 0.6), general plant overheads (f_{po} = 0.65), maintenance (f_{mt} = 0.05), property taxes and insurance (f_{ti} = 0.02), rent of land (f_{rl} = 0.02) and environmental charges (f_{ec} = 0.01).

$$\mathrm{FOC} = \mathrm{CL}\,(1 + f_{sup})\,\Big((1 + f_{so})\,(1 + f_{po}) + \mathrm{ISBL}\,(f_{mt} + f_{ti})\Big) + \mathrm{FCC}\,(f_{rl} + f_{ec}) \tag{7}$$

Cost data was adapted with the Chemical Engineering plant cost index (CEPCI) whenever not directly available, such that all values of the TEA are expressed in USD 2022. The cost parameters used in the VOC are shown in **Table S15**.

The life cycle assessment (LCA) was carried out following the ISO 14040/14044 framework.[4, 5] The calculations and the LCI of the foreground system (i.e. all mass and energy input sand outputs of the process) were generated in Brightway v2.4.3[6] using background data from ecoinvent v3.10[7]. The global warming potential (GWP) of the PP hydrogenolysis process for different catalysts and energy sources was assessed using the IPCC 2021 method for climate change, taking into consideration short-lived climate forces and greenhouse gas emissions (including hydrogen and biogenic CO_2) over a timespan of 100 years.

The purpose of the analysis is to determine the life cycle impacts associated to one kilogram of the product mixture obtained from the PP hydrogenolysis process under different experimental scenarios, such that the inventories were defined for the production of multi-product systems, taking into

consideration the extraction of raw materials and the different production steps. These results were benchmarked with the life cycle impact of the petrochemical product mixture that substitutes the hydrogenolysis products in the global market, where their production from fossil feedstock as well as the end-of-life fate of the otherwise unused PP feedstock (here, it was assumed that 30 % of the waste polymer is incinerated and 70 % landfilled) were taken into account. The LCI data used in the assessments are shown in **Tables S16-S18.**

A sensitivity analysis for the commercial PP hydrogenolysis using wind electricity with respect to GWP and TAC was performed to evaluate the robustness and reliability of the final results and to identify the most influential input parameters that drive those results. These results were compared to the possible range of GWP of the BAU case, derived from a Monte Carlo analysis and to the estimated range of revenues, derived from minimum and maximum market prices of the products over a time span of 2019-2023. The results can be seen in **Tables S23**,**S24**.

Supporting Tables

Table S1. Chemical composition of TiO_2-supported Co_xRu_y catalysts.[a]

Catalyst	Molar content / mol%			Weight content / wt%		
	Ru	Co	Combined	Ru	Co	Combined
Ru_{100}	3.3	0	3.3	4.2	0.0	4.2
$Co_{10}Ru_{90}$	6.2	0.5	6.7	7.7	0.4	8.1
$Co_{10}Ru_{90}$	2.9	0.3	3.2	3.7	0.2	3.9
$Co_{10}Ru_{90}$	1.4	0.2	1.6	1.5	0.1	1.6
$Co_{10}Ru_{90}$	0.7	0.1	0.8	1.0	0.04	1.04
$Co_{30}Ru_{70}$	2.0	0.6	2.6	2.5	0.4	2.9
$Co_{50}Ru_{50}$	1.8	1.3	3.1	2.3	1.0	3.3
$Co_{70}Ru_{30}$	1.1	1.8	2.9	1.3	1.3	2.6
$Co_{90}Ru_{10}$	0.3	2.2	2.5	0.4	1.7	2.1
Co_{100}	0	2.6	2.6	0.0	2.0	2.0

[a] Measured on reduced samples by XRF.

Table S2. Catalytic performance of TiO_2-supported Co_xRu_y catalysts. Reaction conditions: 0.1 g catalyst, 1.0 g PP_{12}, 513 K, 20 bar, 750 rpm, 12 h.

Catalyst	Metal content / mol%	Conversion / %	Yield / %						
			Methane	C_2-C_5	C_6-C_{10}	C_{11}-C_{20}	C_{21}-C_{35}	C_{11+}	Residue
Ru_{100}	3.3	100	58	13	25	3	0	4	0
$Co_{10}Ru_{90}$	6.7	73	8	1	25	32	7	38	27
$Co_{10}Ru_{90}$	3.2	87	11	2	19	32	23	55	13
$Co_{10}Ru_{90}$	1.6	85	5	1	25	37	18	55	15
$Co_{10}Ru_{90}$	0.8	62	3	3	23	28	5	33	38
$Co_{30}Ru_{70}$	2.6	58	1	1	15	26	15	40	42
$Co_{50}Ru_{50}$	3.1	45	2	3	7	13	20	33	55
$Co_{70}Ru_{30}$	2.9	31	1	1	9	15	5	20	69
$Co_{90}Ru_{10}$	2.5	15	1	3	2	4	4	9	85
Co_{100}	2.6	12	1	3	1	4	3	7	88
TiO_2	0	0	0	0	0	0	0	0	100

Table S3. Summary of catalytic performance for polypropylene hydrogenolysis to fuels, comparing literature reports with this work.

Catalyst	Feedstock	Conversion / %	Yield / %		Ref.
			C_{6+}	C_{11+}	
Ru/TiO_2	PP_{250K}	94	66	N.A.[a]	[8]
Ru/TiO_2	PP_{12K}	100	31	29	[9]
Ru/SiO_2	PP_{12K}	35	15	13	[9]
Ru/CeO_2	PP_{12K}	98	17	16	[9]
$NiMoS_x/HY$	PP_{12K}	100	66	15	[10]
$NiMoS_x/HY$	PP_{250K}	100	40	2	[10]
0.5% Ru/C	PP_{250K}	51	46	N.A.	[11]
2% Ru/C	PP_{250K}	82	60	N.A.	[11]
$Co_{10}Ru_{90}$	PP_{12K}	87	74	55	This work
$Co_{10}Ru_{90}$	PP_{250K}	82	65	40	This work

[a] Not available.

Table S4. Comparison of the catalytic performance of TiO_2-supported $Co_{10}Ru_{90}$ (this work) and Ru_1/Co-LDH SAA catalyst[12] with comparable Ru contents.

Catalyst	Metal content / mol%		Plastic	Conversion / %	Yield / %					
	Co	Ru			Methane	C_2-C_5	C_6-C_{10}	C_{11}-C_{20}	C_{21}-C_{35}	C_{6+}
$Co_{10}Ru_{90}$[a]	0.1	1.5	PP_{12K}	85	5	1	25	37	18	80
Ru_1/Co-LDH[a]	49.1	1.3	PP_{12K}	100	33	13	32	15	7	54
Ru_1/Co-LDH[b,c]	49.1	1.3	PP_{4K}	98	7	1	15	53	22	90

[a] Reaction conditions: 0.1 g catalyst, 1.0 g polymer, 513 K, 20 bar, 750 rpm, 12 h.

[b] Reaction conditions: 0.05 g catalyst, 1.0 g polymer, 533 K, 20 bar, 200 rpm, 5 h.

[c] Performance data were estimated based on the product distribution reported in Fig. 8.

Table S5. Catalytic performance of MO_x-supported $Co_{10}Ru_{90}$ catalysts. Reaction conditions: 0.1 g catalyst, 1.0 g PP_{12}, 513 K, 20 bar, 750 rpm, 12 h.

MO_x	Conversion / %	Yield / %						
		Methane	C_2-C_5	C_6-C_{10}	C_{11}-C_{20}	C_{21}-C_{35}	C_{11+}	Residue
SiO_2	13	4	1	4	3	1	4	87
Al_2O_3	98	18	6	35	27	12	39	2
ZrO_2	69	4	2	26	22	16	38	31
TiO_2	87	11	2	19	32	23	55	13
CeO_2	48	6	2	18	16	6	22	52

Table S6. Catalytic performance of TiO_2-supported $Co_{10}Ru_{90}$ over multiple consecutive runs. Reaction conditions: 0.1 g catalyst, 1.0 g PP_{12}, 513 K, 20 bar, 750 rpm, 12 h.

Run	Conversion / %	Yield / %						
		Methane	C_2-C_5	C_6-C_{10}	C_{11}-C_{20}	C_{21}-C_{35}	C_{11+}	Residue
1	87	11	2	19	32	23	55	13
2	87	8	3	19	50	7	57	13
3	93	14	4	19	49	7	56	7
4	87	8	2	15	52	11	63	13

Table S7. Catalytic performance of TiO_2-supported Ru_{100} and $Co_{10}Ru_{90}$ catalysts for different PP-based materials. Reaction conditions: 0.1 g catalyst, 1.0 g polymer, 513 K, 20 bar, 750 rpm.

Material	Catalyst	*t* / h	Conversion / %	Yield / %						
				Methane	C_2-C_5	C_6-C_{10}	C_{11}-C_{20}	C_{21}-C_{35}	C_{11+}	Residue
	Ru_{100}	12	100	58	13	25	3	0	4	0
PP_{12}	$Co_{10}Ru_{90}$	12	87	11	2	19	32	23	55	13
	$Co_{10}Ru_{90}$	24	100	18	6	18	26	33	59	0
	Ru_{100}	12	100	73	22	5	0	0	0	0
PP_{250}	$Co_{10}Ru_{90}$	12	64	5	2	21	25	12	36	36
	$Co_{10}Ru_{90}$	24	82	17	2	24	32	9	40	18
	Ru_{100}	12	88	59	21	4	4	1	5	12
PP bottle	$Co_{10}Ru_{90}$	12	54	7	1	25	19	2	21	46
	$Co_{10}Ru_{90}$	24	100	25	4	45	25	2	27	0
	Ru_{100}	12	100	64	19	8	7	2	9	0
Shampoo cap	$Co_{10}Ru_{90}$	12	32	2	1	6	13	9	22	68
	$Co_{10}Ru_{90}$	24	80	3	1	32	39	5	44	20
	Ru_{100}	12	92	64	18	4	4	2	6	8
Yogurt cup	$Co_{10}Ru_{90}$	12	53	3	1	11	19	19	38	47
	$Co_{10}Ru_{90}$	24	62	7	1	26	24	4	28	38

Table S8. EXAFS fitting results for TiO_2-supported Co_xRu_y catalysts with total metal content of 3 mol%.

Catalyst	K edge	Shell	R / Å	Coordination number / -	σ^2 / Å^2
Ru_{100}	Ru	Ru-Ru	2.67 ± 0.01	7.0 ± 0.6	0.004 ± 0.001
$Co_{10}Ru_{90}$	Ru	Ru-Ru	2.66 ± 0.01	6.3 ± 0.8	0.003 ± 0.002
	Ru	Ru-Co	2.56 ± 0.01	0.6 ± 0.2	0.006 ± 0.006
	Co	Co-Ru	2.55 ± 0.01	4.1 ± 0.7	0.006 ± 0.004
	Co	Co-Co	N.D.[a]	N.D.	N.D.
$Co_{30}Ru_{70}$	Ru	Ru-Ru	2.65 ± 0.01	3.5 ± 0.6	0.004 ± 0.002
	Ru	Ru-Co	2.56 ± 0.01	3.6 ± 0.7	0.003 ± 0.002
	Co	Co-Ru	2.55 ± 0.01	3.5 ± 0.3	0.005 ± 0.002
	Co	Co-Co	2.49 ± 0.02	1.5 ± 0.4	0.004 ± 0.003
$Co_{50}Ru_{50}$	Ru	Ru-Ru	2.65 ± 0.02	3.2 ± 0.5	0.004 ± 0.002
	Ru	Ru-Co	2.55 ± 0.01	4.2 ± 0.8	0.004 ± 0.003
	Co	Co-Ru	2.56 ± 0.02	3.6 ± 0.6	0.006 ± 0.002
	Co	Co-Co	2.51 ± 0.01	2.4 ± 0.4	0.002 ± 0.002
$Co_{70}Ru_{30}$	Ru	Ru-Ru	2.64 ± 0.01	3.0 ± 0.3	0.004 ± 0.002
	Ru	Ru-Co	2.55 ± 0.01	4.8 ± 0.2	0.003 ± 0.003
	Co	Co-Ru	2.56 ± 0.02	2.8 ± 0.2	0.005 ± 0.002
	Co	Co-Co	2.51 ± 0.01	4.2 ± 0.3	0.006 ± 0.003
$Co_{90}Ru_{10}$	Co	Co-Ru	2.56 ± 0.02	0.6 ± 0.4	0.005 ± 0.002
	Co	Co-Co	2.49 ± 0.01	6.6 ± 0.8	0.004 ± 0.001
Co_{100}	Co	Co-Co	2.49 ± 0.01	7.1 ± 0.2	0.004 ± 0.001

[a] Not detected.

Table S9. EXAFS fitting results for TiO_2-supported $Co_{10}Ru_{90}$ catalysts with different total metal contents.

Content / mol%	K edge	Shell	R / Å	Coordination number / -	σ^2 / Å^2
6.7	Ru	Ru-Ru	2.67 ± 0.01	7.1 ± 0.7	0.003 ± 0.001
	Ru	Ru-Co	2.56 ± 0.01	0.5 ± 0.2	0.007 ± 0.007
	Co	Co-Ru	2.55 ± 0.02	4.3 ± 0.6	0.003 ± 0.001
	Co	Co-Co	N.D.[a]	N.D.	N.D.
	Co	Co-O	2.01 ± 0.12	0.8 ± 0.2	0.007 ± 0.003
3.2	Ru	Ru-Ru	2.66 ± 0.01	6.3 ± 0.8	0.003 ± 0.002
	Ru	Ru-Co	2.56 ± 0.01	0.6 ± 0.2	0.006 ± 0.006
	Co	Co-Ru	2.55 ± 0.01	4.1 ± 0.7	0.006 ± 0.004
	Co	Co-Co	N.D.	N.D.	N.D.
1.6	Ru	Ru-Ru	2.67 ± 0.02	5.9 ± 1.2	0.004 ± 0.002
	Ru	Ru-Co	2.55 ± 0.02	0.4 ± 0.3	0.003 ± 0.003
0.8	Ru	Ru-Ru	2.67 ± 0.02	4.2 ± 0.7	0.004 ± 0.001
	Ru	Ru-Co	N.D.	N.D.	N.D.

[a] Not detected.

Table S10. Catalytic performance of TiO_2-supported $Co_{10}Ru_{90}$ at different reaction times. Reaction conditions: 0.1 g catalyst, 1.0 g PP_{12}, 513 K, 20 bar, 750 rpm.

Time / h	Conversion / %	Yield / %						
		Methane	C_2-C_5	C_6-C_{10}	C_{11}-C_{20}	C_{21}-C_{35}	C_{11+}	Residue
1	28	0	0	4	13	11	24	72
6	55	2	1	14	16	22	39	45
12	87	11	2	19	32	23	55	13
18	92	11	4	34	33	10	43	8
24	100	16	7	18	26	33	59	0

Table S11. Catalytic performance of TiO_2-supported Ru_{100} catalyst at different reaction times. Reaction conditions: 0.1 g catalyst, 1.0 g PP_{12}, 513 K, 20 bar, 750 rpm.

Time / h	Conversion / %	Yield / %						
		Methane	C_2-C_5	C_6-C_{10}	C_{11}-C_{20}	C_{21}-C_{35}	C_{11+}	Residue
0.2	27	0	0	14	11	2	13	73
1	40	4	3	11	14	8	22	60
6	66	10	5	17	25	9	33	34
12	100	58	13	25	3	0	4	0

Table S12. Catalytic performance of TiO_2-supported $Co_{10}Ru_{90}$ for different virgin polyolefins. Reaction conditions: 0.1 g catalyst, 1.0 g polymer, 513 K, 20 bar, 750 rpm, 12 h.

Polymer	Conversion / %	Yield / %						
		Methane	C_2-C_5	C_6-C_{10}	C_{11}-C_{20}	C_{21}-C_{35}	C_{11+}	Residue
PP_{12}	87	11	2	19	32	23	55	13
$HDPE_{100}$	100	77	18	4	2	0	2	0

Table S13. Parameters a, fixed cost parameter; b, variable cost parameter; and n, scaling factor used in equation (2) to calculate the purchase equipment cost (C) per type of equipment in kUSD.

Equipment type	a	b	n	Reference
Coal gasifier	0	178000[a]	0.67	Martelli et al.[13]
Plate and frame filter	110000	77000	0.50	Sinnott and Towler[3]
Centrifugal compressor	490000	16800	0.60	Sinnott and Towler[3]
Single stage centrifugal pump	6900	206	0.90	Sinnott and Towler[3]
Pressure-swing-adsorption	0	7790[b]	0.65	Onel et al.[14]
Vertical pressure vessel	10000	29	0.85	Sinnott and Towler[3]
Pressure vessel	10000	29	0.85	Sinnott and Towler[3]
Sieve trays (1 tray)	110	380	1.80	Sinnott and Towler[3]
U-tube shell and tube heat exchanger	24000	46	1.20	Sinnott and Towler[3]

[a] Alternative sizing equation was used for the gasifier based on Martelli *et al.*[13] (for a standard gasifier with SG coolers).[b] Alternative sizing equation was used for the PSA based on Onel *et al.*[14] and adjusted to kUSD 2010 - unit at which the coefficients are described in Sinnott and Towler[3] - for consistency purposes.

Table S14. Variables used for the annualized capital cost (ACC) calculation.

Variable	Value	Unit
Annual hours of operation	8000.0	h y^{-1}
Plant lifetime	25.0	y
Interest rate	10.0	%
CEPCI[a] 2010	550.8	USD
CEPCI 2022	816.0	USD

[a] The Chemical Engineering Plant Cost Index, CEPCI is used to account for inflation over the years and adequately adjust the capital expenditures, CAPEX, here calculated with cost correlations from Sinnott & Towler that provide costs in USD 2010.[3]

Table S15. Cost parameters considered for the operation expenditure (OPEX) calculations in the techno-economic assessment. The table includes costs of raw materials, electricity, heating and cooling utilities used in the process shown in **Figure S18**. All costs are reported in terms of USD unit^{-1}, for which the unit matches the units of the life cycle inventories (**Table S16**).

Item	Cost avg. / USD unit^{-1}	Reference
Waste PP	0.30	Salah *et al.*[2]
Hydrogen, SMR	1.26	Nabera *et al.*[15]
Hydrogen, wind off-shore	6.21	Nabera *et al.*[15]
Hydrogen, solar PV	8.87	Nabera *et al.*[15]
n-Hexane solvent	1.14	ChemAnalyst[16]
Ru_{100} catalyst	0.69	Statista[a]
$Co_{10}Ru_{90}$ catalyst	0.61	Statista[a]
Cooling water at 20-35ºC	3.23×10^{-4}	Ioannou *et al.*[17]
Cryogenic cooling at −25ºC	1.62×10^{-2}	Ioannou *et al.*[17]
Cryogenic cooling −125ºC	7.45×10^{-2}	Ioannou *et al.*[17]
Heating utility, natural gas	5.67×10^{-2}	World Bank[18],[b]
Electricity	0.07	Turton *et al.*[19]

[a] The catalyst price was calculated based on the market prices for ruthenium (16.45 USD g^{-1})[20], cobalt (0.027 USD g^{-1})[21], titania (0.001 USD g^{-1})[22]. [b] The heating utility cost was derived from the global average cost of natural gas in 2019.

Table S16. Life cycle inventory (LCI) of the hydrogenolysis of PP process, shown in **Figure S18**. Mass and energy flows were obtained from the process flowsheet, normalized per kilogram of product generated and vary depending on the plastic type.

Material/Energy Flow	Unit	Refence LCI
Products		
Methane	kg	Ecoinvent 3.10[7]
Light gases, C_2-C_5	kg	Ecoinvent 3.10[7]
Gasoline, C_6-C_{12}	kg	Ecoinvent 3.10[7]
Diesel, C_{13}-C_{20}	kg	Ecoinvent 3.10[7]
Motor oil, C_{21}-C_{45}	kg	Ecoinvent 3.10[7]
Technosphere flows		
Waste PP	kg	Salah et al., 2023[2]
Hydrogen	kg	Premise[23]
Cooling water at 20-35 ºC	MJ	Ioannou et al., 2023[17]
Cryogenic cooling at −25 ºC	MJ	Ioannou et al., 2023[17]
Cryogenic cooling at −125 ºC	MJ	Ioannou et al., 2023[17]
Heating utility, natural gas	MJ	Ecoinvent 3.10[7]
Electricity	kWh	Ecoinvent 3.10[7]
Ru_{100} catalyst	kg	**Table S17**
$Co_{10}Ru_{90}$ catalyst	kg	**Table S17**
n-Hexane solvent	kg	Ecoinvent 3.10[7]

Table S17. Life cycle inventory (LCI) of the catalysts.

Material/Energy Flow	Amount	Unit	Reference LCI
Product			
Ru_{100} catalyst	1.000	kg	
Exchanges with the technosphere			
Titanium	0.958	kg	Ecoinvent 3.10[7]
Ruthenium	0.042	kg	Ecoinvent 3.10[7],a
Product			
$Co_{10}Ru_{90}$ catalyst	1.000	kg	
Exchanges with the technosphere			
Titanium	0.961	kg	Ecoinvent 3.10[7]
Ruthenium	0.037	kg	Ecoinvent 3.10[7],a
Cobalt	0.002	kg	Ecoinvent 3.10[7]
Product			

[a] Ruthenium is a by-product of platinum production. Ecoinvent applies economic allocation to all metals of the platinum metals group.[23] Thus, all flows from the Ecoinvent 3.10 activity relative to the production of 1 kg of platinum were divided by the price of platinum and multiplied by the price of ruthenium to obtain the LCI relative to the production of 1 kg of ruthenium.

Table S18. Reference Ecoinvent 3.10[7] and Premise[24] background data used as input flows to LCIs described in **Table S16**,**S17** under "Technosphere flows".

Material/Energy Flow	Reference product	Activity name	Location
Ecoinvent 3.10[7] activities			
Methane	market for natural gas, high pressure	market for natural gas, high pressure	RoW
Light gases, C_2-C_5	butane	natural gas production	RoW
Gasoline, C_6-C_{12}	light fuel oil	light fuel oil production, petroleum refinery operation	RoW
Diesel, C_{13}-C_{20}	diesel	diesel production, petroleum refinery operation	RoW
Motor oil, C_{21}-C_{45}	heavy fuel oil	heavy fuel oil production, petroleum refinery operation	RoW
PP primary production	PP, granulate	PP production, granulate	RoW
PP incineration	waste PP	treatment of waste PP, municipal incineration	RoW
PP landfilling	waste PP	treatment of waste polyepropylene, sanitary landfill	RoW
Heating utility, natural gas	heat, district or industrial, natural gas	market group for heat, district or industrial, natural gas	GLO
Electricity	electricity, high voltage	market group for electricity, high voltage	GLO
n-Hexane solvent	hexane	market for hexane	GLO
Titanium	titanium	market for titanium	GLO
Ruthenium - adapted from:	platinum	platinum group metal, extraction and refinery operations	ZA
Cobalt	cobalt	market for cobalt	GLO
Premise[24] activities			
Hydrogen - fossil	hydrogen, gaseous, 25 bar	hydrogen production, steam methane reforming of natural gas, 25 bar	World
Hydrogen - wind	hydrogen, gaseous, 30 bar	hydrogen production, gaseous, 30 bar, from PEM electrolysis, from offshore wind electricity	RoW
Hydrogen - solar	hydrogen, gaseous, 30 bar	hydrogen production, gaseous, 30 bar, from PEM electrolysis, from solar PV electricity	RoW

Table S19. Breakdown of revenues and total annualized costs for the hydrogenolysis of PP_{12} when using $Co_{10}Ru_{90}$ as catalyst at different reaction times (**Table S10**) and different sources of hydrogen.

Contribution	1 h / USD $kg_{product}^{-1}$			6 h / USD $kg_{product}^{-1}$			12 h / USD $kg_{product}^{-1}$			18 h / USD $kg_{product}$			24 h / USD $kg_{product}$		
	Fossi	Wind	Solar	Fossil	Wind	Solar	Fossil	Wind	Solar	Fossil	Wind	Solar	Fossil	Wind	Solar
Revenues															
Methane	0.00	0.00	0.00	0.01	0.01	0.01	0.03	0.03	0.03	0.03	0.03	0.03	0.06	0.06	0.06
Light gases	0.00	0.00	0.00	0.00	0.00	0.00	0.01	0.01	0.01	0.02	0.02	0.02	0.02	0.02	0.02
Gasoline	0.05	0.05	0.05	0.20	0.20	0.20	0.27	0.27	0.27	0.48	0.48	0.48	0.25	0.25	0.25
Diesel	0.20	0.20	0.20	0.25	0.25	0.25	0.49	0.49	0.49	0.50	0.50	0.50	0.39	0.39	0.39
Motor oil	0.20	0.20	0.20	0.40	0.40	0.40	0.41	0.41	0.41	0.17	0.17	0.17	0.64	0.64	0.64
Residue	0.00	0.00	0.00	0.00	0.00	0.00	0.00	0.00	0.00	0.00	0.00	0.00	0.00	0.00	0.00
Total	0.46	0.46	0.46	0.86	0.86	0.86	1.21	1.21	1.21	1.21	1.21	1.21	1.36	1.36	1.36
Total annualized cost															
CAPEX	0.06	0.06	0.06	0.07	0.07	0.07	0.09	0.09	0.09	0.09	0.09	0.09	0.10	0.10	0.10
Catalyst	0.30	0.30	0.30	0.30	0.30	0.30	0.30	0.30	0.30	0.30	0.30	0.30	0.29	0.29	0.29
FOC	0.00	0.00	0.00	0.00	0.00	0.00	0.01	0.01	0.01	0.01	0.01	0.01	0.01	0.01	0.01
H_2	0.00	0.02	0.02	0.01	0.05	0.07	0.03	0.16	0.22	0.04	0.17	0.25	0.05	0.23	0.33
Utilities	0.02	0.02	0.02	0.02	0.02	0.02	0.03	0.03	0.03	0.03	0.03	0.03	0.04	0.04	0.04
wPP	0.30	0.30	0.30	0.29	0.29	0.29	0.29	0.29	0.29	0.29	0.29	0.29	0.29	0.29	0.29
Total	0.68	0.69	0.70	0.69	0.73	0.76	0.74	0.86	0.93	0.75	0.88	0.96	0.78	0.96	1.06

Table S20. Contributions to the global warming potential (GWP_{CR}) from a cradle-to-grave life cycle assessment of hydrogenolysis of PP_{12} when using $Co_{10}Ru_{90}$ as catalyst at different reaction times (**Table S10**). The GWP of the equivalent business-as-usual process (GWP_{BAU}) were provided for reference.

Contribution	1 h / kg_{CO2} $kg_{product}^{-1}$			6 h / kg_{CO2} $kg_{product}^{-1}$			12 h / kg_{CO2}			18 h / kg_{CO2}			24 h / kg_{CO2}		
	Foss	Wind	Solar	Fossil	Wind	Solar	Fossi	Wind	Solar	Fossi	Wind	Solar	Fossi	Wind	Solar
GWP_{BAU}															
Methane	0.02	0.02	0.02	0.13	0.13	0.13	0.68	0.68	0.68	0.69	0.69	0.69	1.12	1.12	1.12
Light gases	0.01	0.01	0.01	0.05	0.05	0.05	0.13	0.13	0.13	0.24	0.24	0.24	0.31	0.31	0.31
Gasoline	0.21	0.21	0.21	0.79	0.79	0.79	1.06	1.06	1.06	1.88	1.88	1.88	0.97	0.97	0.97
Diesel	0.71	0.71	0.71	0.92	0.92	0.92	1.75	1.75	1.75	1.80	1.80	1.80	1.37	1.37	1.37
Motor oil	0.56	0.56	0.56	1.09	1.09	1.09	1.11	1.11	1.11	0.45	0.45	0.45	1.71	1.71	1.71
Residue	4.62	4.62	4.62	2.94	2.94	2.94	0.91	0.91	0.91	0.56	0.56	0.56	0.00	0.00	0.00
Total	6.14	6.14	6.14	5.93	5.93	5.93	5.64	5.64	5.64	5.61	5.61	5.61	5.48	5.48	5.48
GWP_{CR}															
wPP	3.39	3.39	3.39	3.37	3.37	3.37	3.31	3.31	3.31	3.30	3.30	3.30	3.27	3.27	3.27
Ru catalyst	0.00	0.00	0.00	0.00	0.00	0.00	0.00	0.00	0.00	0.00	0.00	0.00	0.00	0.00	0.00
RuCo catalyst	0.89	0.89	0.89	0.89	0.89	0.89	0.87	0.87	0.87	0.87	0.87	0.87	0.86	0.86	0.86
H_2	0.03	0.01	0.02	0.09	0.01	0.06	0.28	0.04	0.17	0.31	0.04	0.19	0.41	0.06	0.26
Utilities	0.12	0.12	0.12	0.14	0.14	0.14	0.23	0.23	0.23	0.24	0.24	0.24	0.34	0.34	0.34
Total	4.43	4.41	4.42	4.49	4.41	4.45	4.69	4.45	4.59	4.72	4.45	4.61	4.89	4.54	4.74

Table S21. Breakdown of revenues and total annualized costs for the hydrogenolysis of different PP feedstocks when using different sources of hydrogen.

Feedstocks	Contribution	Ru_{100} / USD $kg_{product}^{-1,a}$			$Co_{10}Ru_{90}$ / USD $kg_{product}^{-1,a}$			$Co_{10}Ru_{90}$ / USD $kg_{product}^{-1,b}$		
		Fossil	Wind	Solar	Fossil	Wind	Solar	Fossil	Wind	Solar
PP_{12}	Revenue[c]	0.61	0.61	0.61	1.21	1.21	1.21	1.36	1.36	1.36
	Total annualized cost[d]	0.94	1.41	1.67	0.74	0.86	0.93	0.78	0.96	1.06
PP_{250}	Revenue	0.37	0.37	0.37	0.91	0.91	0.91	1.02	1.02	1.02
	Total annualized cost	0.95	1.51	1.80	0.71	0.78	0.82	0.76	0.92	1.01
PP bottle	Revenue	0.38	0.38	0.38	0.72	0.72	0.72	1.11	1.11	1.11
	Total annualized cost	0.91	1.37	1.62	0.72	0.80	0.85	0.79	1.02	1.15
Shampoo cap	Revenue	0.50	0.50	0.50	0.49	0.49	0.49	1.17	1.17	1.17
	Total annualized cost	0.93	1.43	1.70	0.69	0.72	0.74	0.71	0.78	0.81
Yogurt cup	Revenue	0.40	0.40	0.40	0.82	0.82	0.82	0.83	0.83	0.83
	Total annualized cost	0.93	1.42	1.69	0.70	0.74	0.76	0.72	0.81	0.86

[a] Reaction time: 12 h. [b] Reaction time: 24 h. [c] Revenue was calculated from the sale of all products, including methane, light gases, gasoline, diesel, and motor oil. [d] Total annualized cost was calculated by accounting for CAPEX, catalyst costs, FOC, hydrogen, utilities, and wPP.

Table S22. Contributions to the global warming potential (GWP_{CR}) from a cradle-to-grave life cycle assessment of hydrogenolysis of different PP feedstocks when using different sources of hydrogen. The GWP of the equivalent business-as-usual process (GWP_{BAU}) were provided for reference.

Feedstocks	Contribution	Ru_{100} / kg_{CO2} $kg_{product}^{-1}$,[a]			$Co_{10}Ru_{90}$ / kg_{CO2} $kg_{product}^{-1}$,[a]			$Co_{10}Ru_{90}$ / kg_{CO2} $kg_{product}^{-1}$,[b]		
		Fossil	Wind	Solar	Fossil	Wind	Solar	Fossil	Wind	Solar
PP_{12}	GWP_{BAU}[c]	5.27	5.27	5.27	5.64	5.64	5.64	5.48	5.48	5.48
	GWP_{CR}[d]	5.67	4.75	5.27	4.69	4.45	4.59	4.89	4.54	4.74
PP_{250}	GWP_{BAU}	5.21	5.21	5.21	5.84	5.84	5.84	5.65	5.65	5.65
	GWP_{CR}	5.75	4.68	5.29	4.55	4.41	4.49	4.75	4.43	4.61
PP bottle	GWP_{BAU}	5.36	5.36	5.36	5.90	5.90	5.90	5.47	5.47	5.47
	GWP_{CR}	5.52	4.63	5.14	4.57	4.41	4.50	4.90	4.44	4.70
Shampoo cap	GWP_{BAU}	5.26	5.26	5.26	6.09	6.09	6.09	5.75	5.75	5.75
	GWP_{CR}	5.60	4.65	5.19	4.46	4.40	4.43	4.53	4.40	4.48
Yogurt cup	GWP_{BAU}	5.31	5.31	5.31	5.93	5.93	5.93	5.85	5.85	5.85
	GWP_{CR}	5.59	4.65	5.18	4.49	4.40	4.45	4.58	4.41	4.51

[a] Reaction time: 12 h. [b] Reaction time: 24 h. [c] GWP_{BAU} was calculated by accounting for methane, light gases, gasoline, diesel, motor oil, and residue. [d] GWP_{CR} was calculated by accounting for catalyst, hydrogen, utilities, and wPP.

Table S23. GWP sensitivity analysis for different PP feedstocks using $Co_{10}Ru_{90}$ and 24 h reaction time. All values are displayed in $kg_{CO2}\ kg_{product}^{-1}$.

Feedstock	Parameter	−25%	−20%	−15%	−10%	−5%	0%	5%	10%	15%	20%	25%
PP bottle	wPP	3.64	3.80	3.96	4.12	4.28	4.44	4.61	4.77	4.93	5.09	5.25
	Catalyst	4.23	4.27	4.32	4.36	4.40	4.44	4.49	4.53	4.57	4.62	4.66
	H_2	4.43	4.43	4.43	4.44	4.44	4.44	4.45	4.45	4.46	4.46	4.46
	Utilities	4.37	4.39	4.40	4.42	4.43	4.44	4.46	4.47	4.49	4.50	4.51
Shampoo cap	wPP	3.56	3.73	3.90	4.07	4.23	4.40	4.57	4.74	4.91	5.07	5.24
	Catalyst	4.18	4.23	4.27	4.31	4.36	4.40	4.45	4.49	4.53	4.58	4.62
	H_2	4.40	4.40	4.40	4.40	4.40	4.40	4.40	4.40	4.41	4.41	4.41
	Utilities	4.37	4.37	4.38	4.39	4.40	4.40	4.41	4.42	4.42	4.43	4.44
Yogurt cup	wPP	3.58	3.74	3.91	4.08	4.24	4.41	4.58	4.74	4.91	5.08	5.25
	Catalyst	4.19	4.23	4.28	4.32	4.37	4.41	4.45	4.50	4.54	4.59	4.63
	H_2	4.40	4.41	4.41	4.41	4.41	4.41	4.41	4.41	4.42	4.42	4.42
	Utilities	4.37	4.38	4.39	4.39	4.40	4.41	4.42	4.43	4.44	4.44	4.45

Table S24. TAC sensitivity analysis for different PP feedstocks using $Co_{10}Ru_{90}$ and 24 h reaction time. All values are displayed in USD $kg_{product}^{-1}$.

Feedstock	Parameter	−25%	−20%	−15%	−10%	−5%	0%	5%	10%	15%	20%	25%
PP bottle	wPP	0.95	0.97	0.98	1.00	1.01	1.02	1.04	1.05	1.07	1.08	1.10
	Catalyst	0.95	0.97	0.98	1.00	1.01	1.02	1.04	1.05	1.07	1.08	1.10
	H_2	0.95	0.96	0.98	0.99	1.01	1.02	1.04	1.05	1.07	1.08	1.10
	FOC	1.02	1.02	1.02	1.02	1.02	1.02	1.03	1.03	1.03	1.03	1.03
	ACC	1.00	1.00	1.01	1.01	1.02	1.02	1.03	1.04	1.04	1.05	1.05
	Utilities	1.02	1.02	1.02	1.02	1.02	1.02	1.03	1.03	1.03	1.03	1.03
Shampoo cap	wPP	0.70	0.72	0.73	0.75	0.76	0.78	0.79	0.81	0.82	0.84	0.85
	Catalyst	0.70	0.72	0.73	0.75	0.76	0.78	0.79	0.81	0.82	0.84	0.85
	H_2	0.76	0.76	0.76	0.77	0.77	0.78	0.78	0.79	0.79	0.79	0.80
	FOC	0.78	0.78	0.78	0.78	0.78	0.78	0.78	0.78	0.78	0.78	0.78
	ACC	0.76	0.76	0.77	0.77	0.77	0.78	0.78	0.78	0.79	0.79	0.80
	Utilities	0.77	0.77	0.77	0.78	0.78	0.78	0.78	0.78	0.78	0.78	0.78
Yogurt cup	wPP	0.74	0.75	0.77	0.78	0.80	0.81	0.83	0.84	0.86	0.87	0.89
	Catalyst	0.74	0.75	0.77	0.78	0.80	0.81	0.83	0.84	0.86	0.87	0.89
	H_2	0.78	0.79	0.80	0.80	0.81	0.81	0.82	0.82	0.83	0.84	0.84
	FOC	0.81	0.81	0.81	0.81	0.81	0.81	0.81	0.81	0.81	0.81	0.81
	ACC	0.79	0.80	0.80	0.80	0.81	0.81	0.82	0.82	0.83	0.83	0.83
	Utilities	0.81	0.81	0.81	0.81	0.81	0.81	0.81	0.82	0.82	0.82	0.82

Supporting Figures

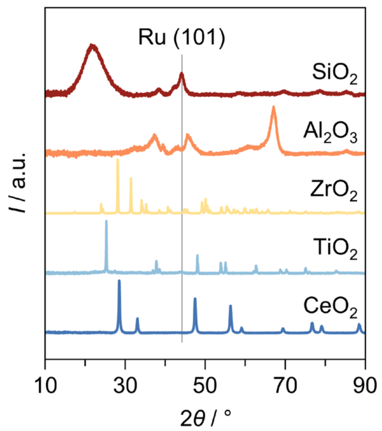

Figure S1. XRD patterns of different MO_x-supported $Co_{10}Ru_{90}$ catalysts with 3 mol% total metal content. The characteristic reflection of Ru (101) facet is indicated.

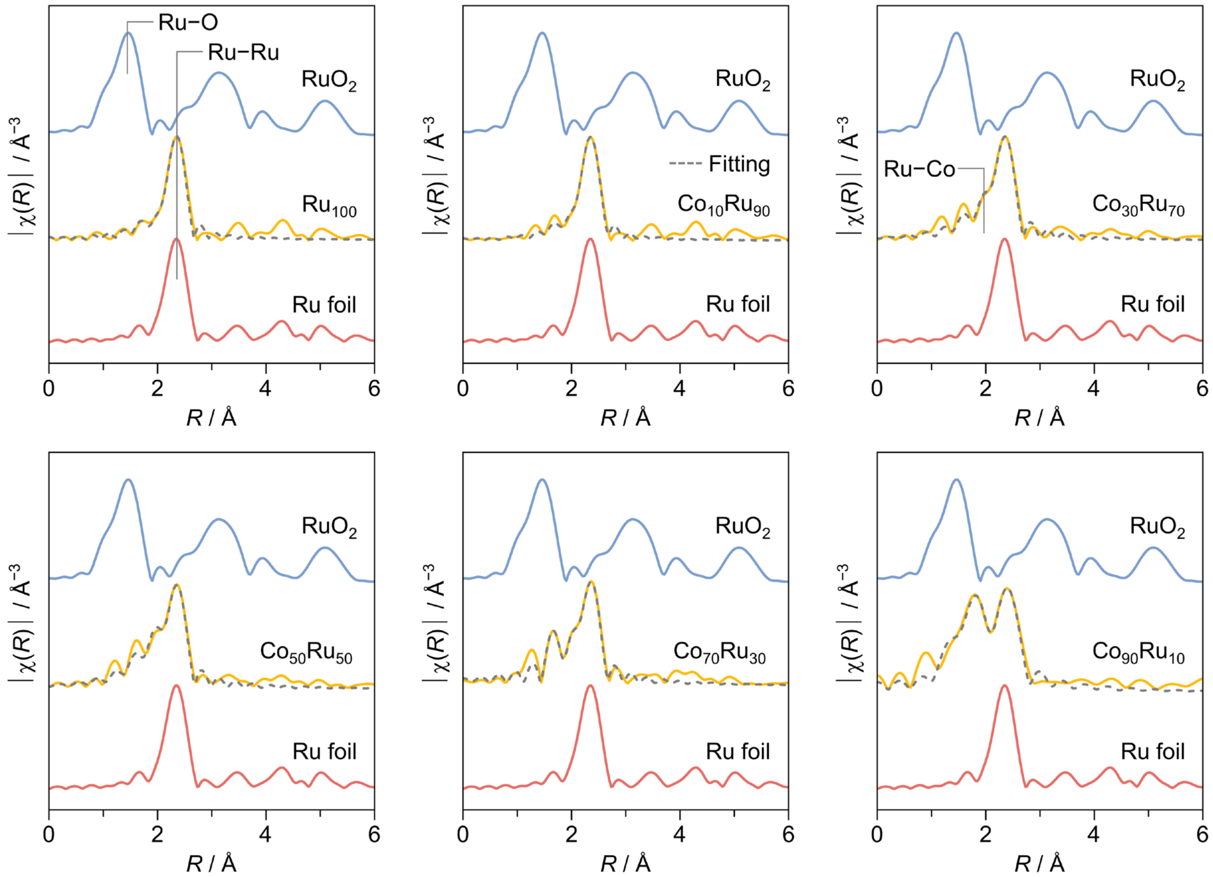

Figure S2. EXAFS profiles and corresponding fittings (dashed lines) of Co_xRu_y catalysts with total metal contents of 3 mol% at Ru K-edge. The profiles of standard RuO_2 and Ru foil were included as references.

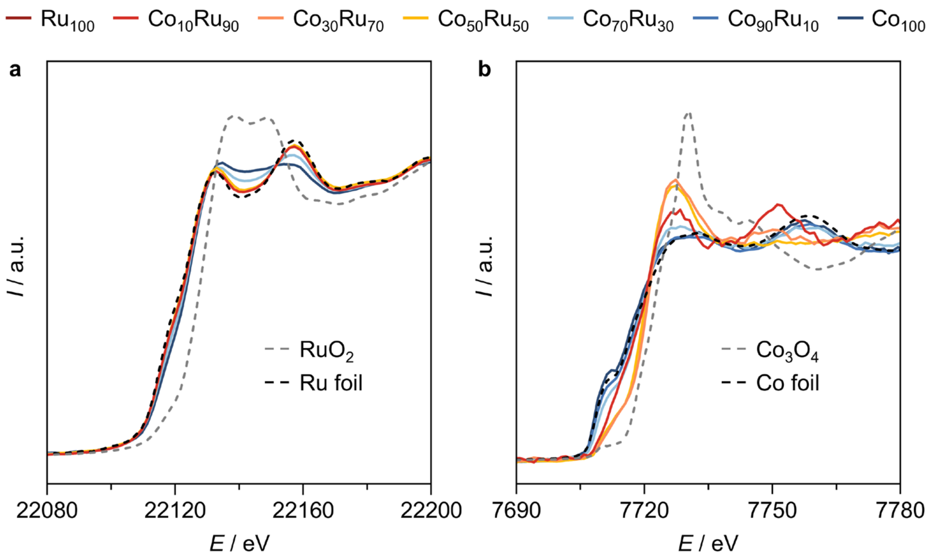

Figure S3. XANES profiles of Co_xRu_y catalysts with total metal contents of 3 mol% at (**a**) Ru K-edge, (**b**) Co K-edge. The profiles of standards (dashed lines) including RuO_2, Ru foil, Co_3O_4, and Co foil were included as references.

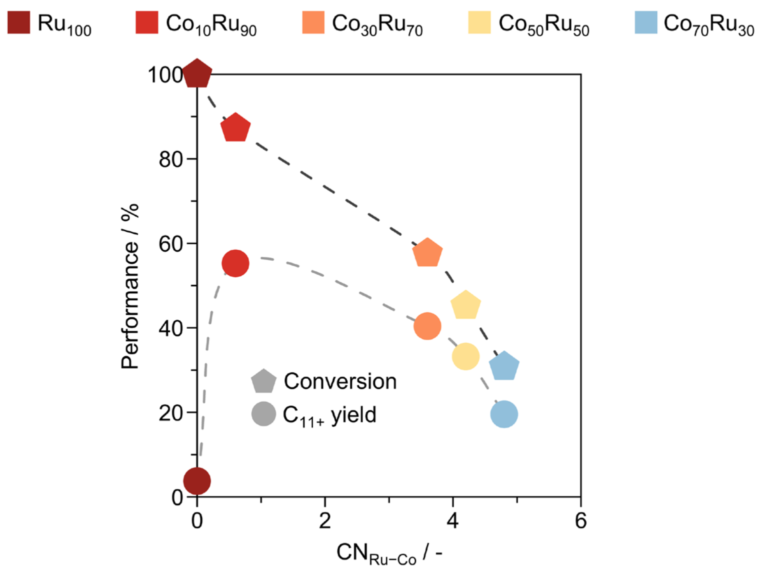

Figure S4. Conversion of PP_{12} and C_{11+} yield as a function of CN_{Ru-Co} for Co_xRu_y catalysts.

a
b
c
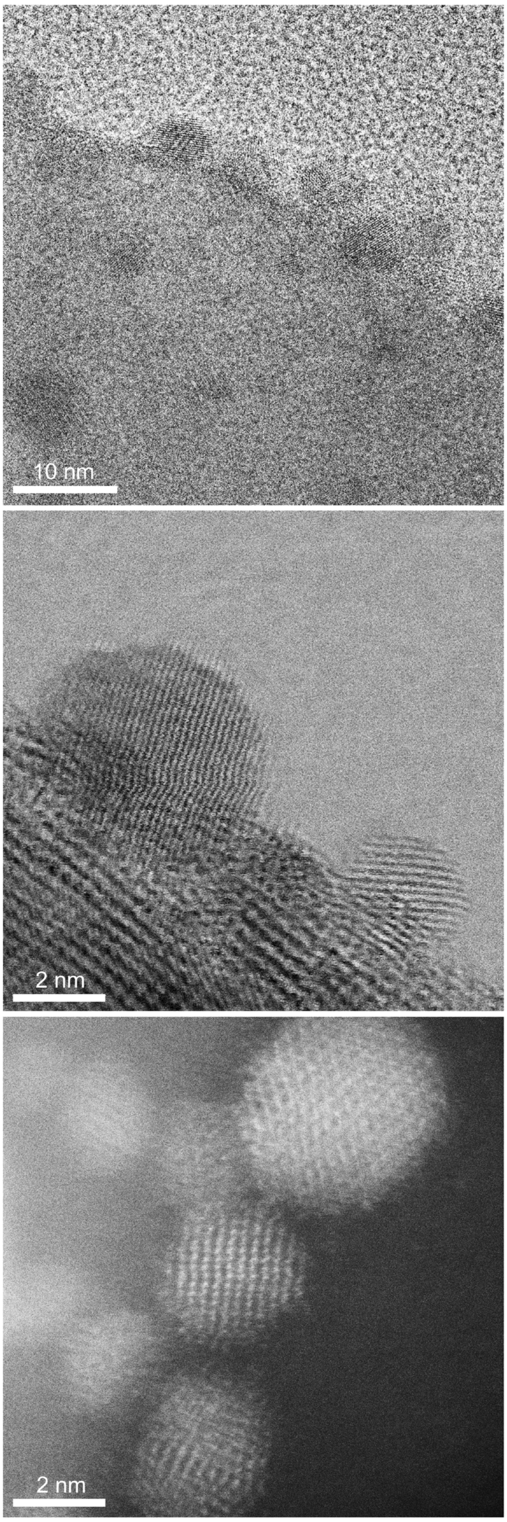

Figure S5. Supplementary microscopy images of $Co_{10}Ru_{90}$ with total metal content of 3 mol%, (**a**) as-prepared, (**b-c**) after reaction.

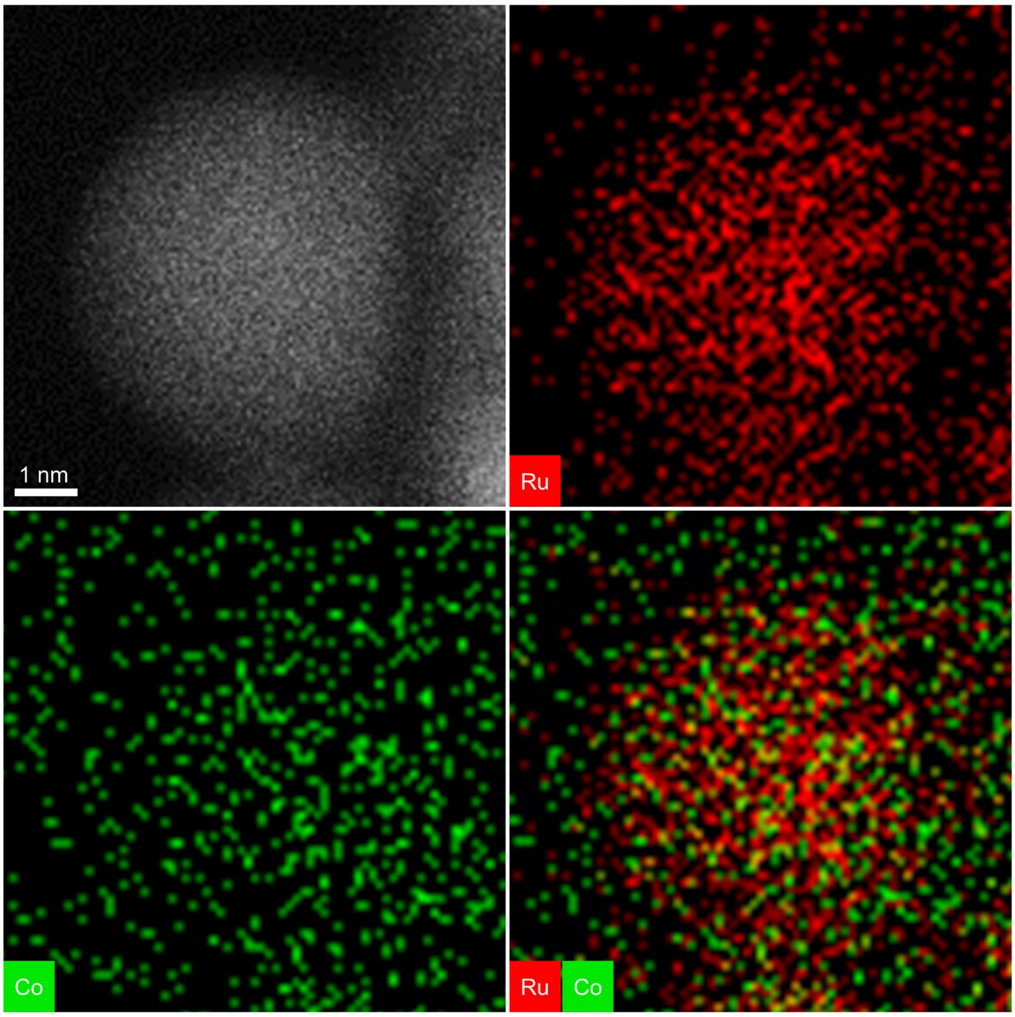

Figure S6. High-resolution STEM image and corresponding EDX elemental mapping of as-prepared TiO_2-supported $Co_{10}Ru_{90}$ catalyst with total metal content of 3.2 mol%.

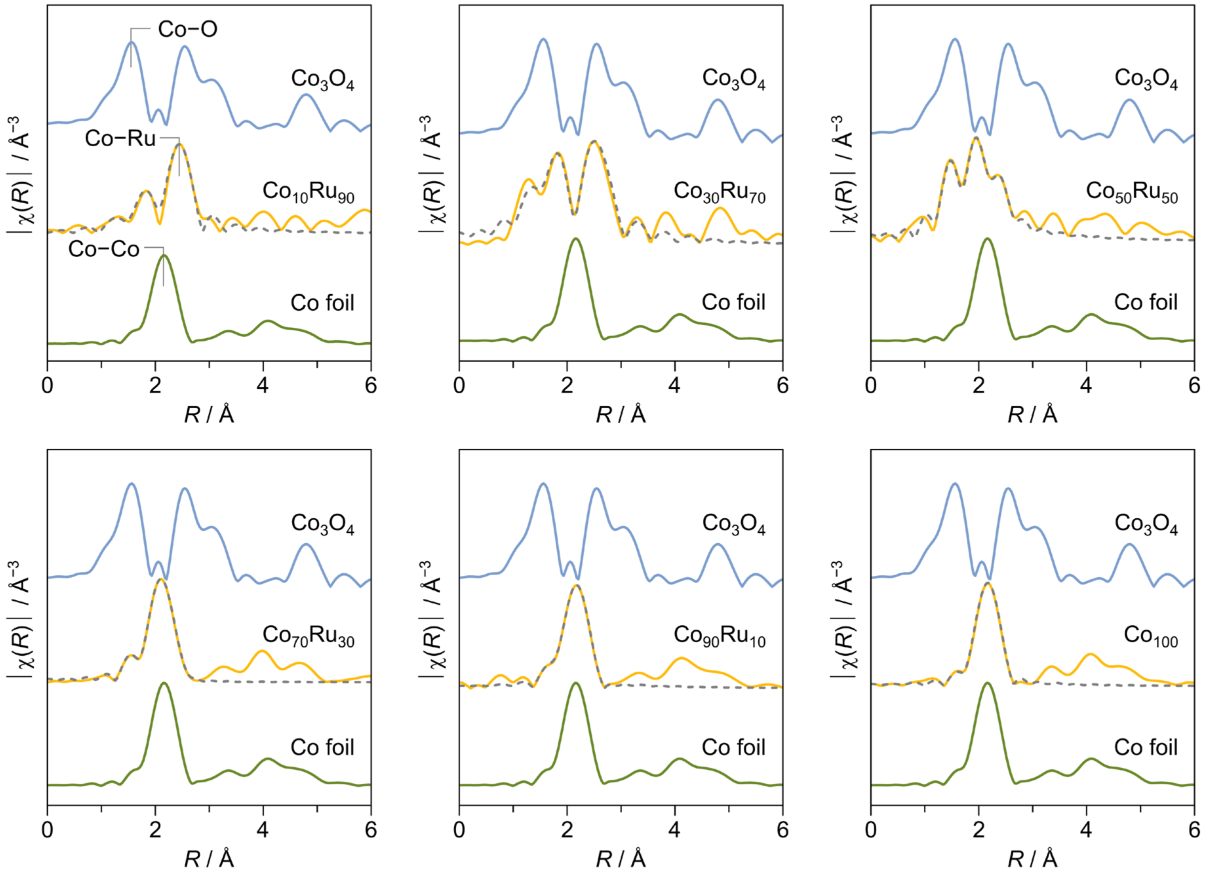

Figure S7. EXAFS profiles and corresponding fittings (dashed lines) of Co_xRu_y catalysts with total metal contents of 3 mol% at Co K-edge. The profiles of standard Co_3O_4 and Co foil were included as references.

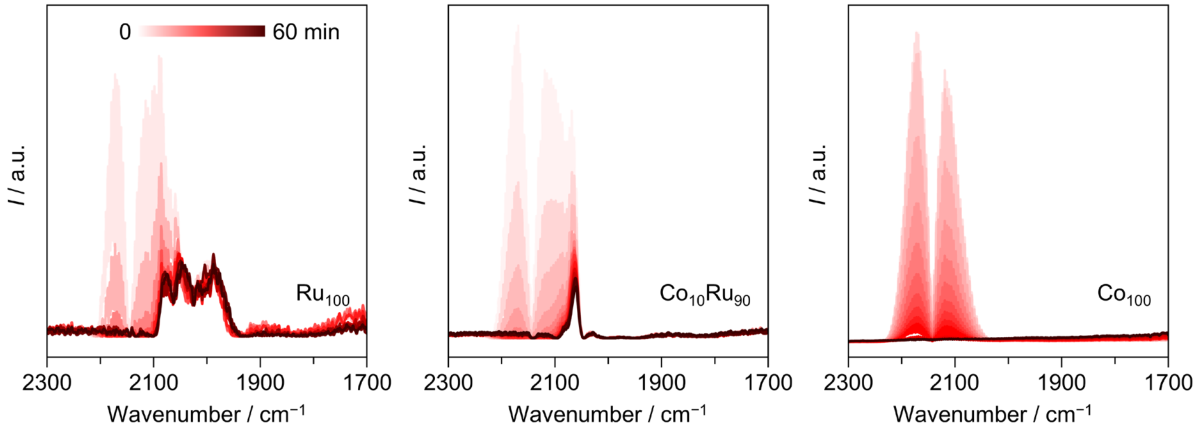

Figure S8. Time-resolved CO desorption profiles.

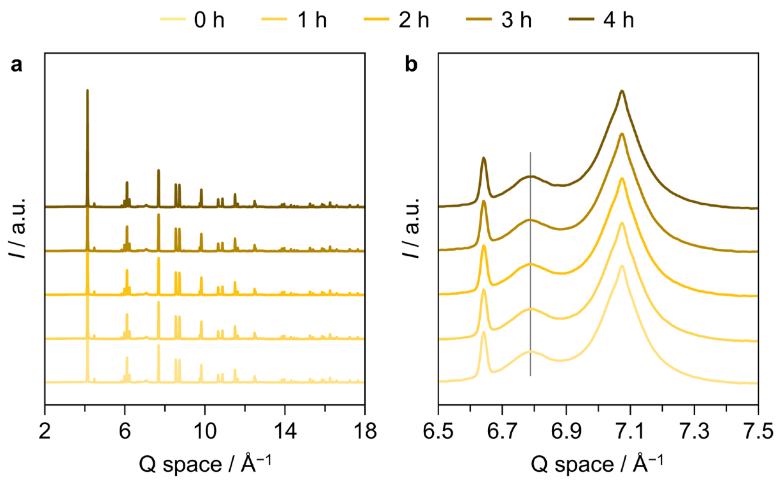

Figure S9. Time-resolved operando HRPD spectra of TiO_2-supported Ru_{100} at (**a**) full and (**b**) enlarged scale. The gray lines indicate the characteristic positions of Ru species. Reaction conditions: PP_{12}:catalyst = 1:1 mass ratio, 513 K, 20 bar H_2, 4 h.

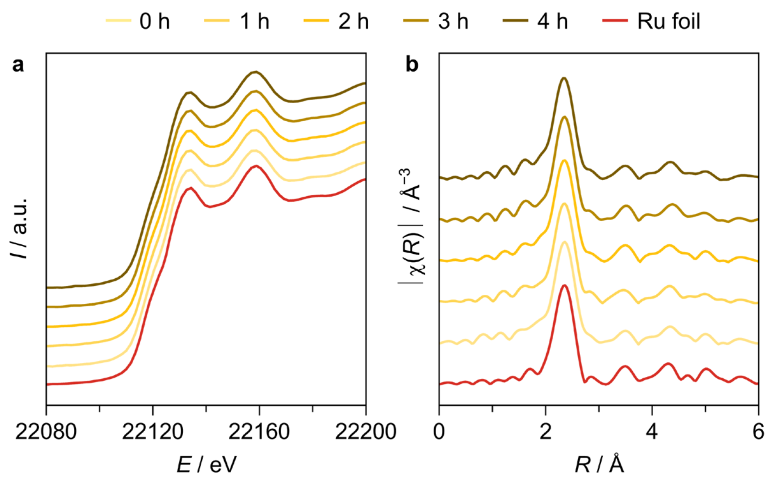

Figure S10. Time-resolved operando (**a**) XANES and (**b**) EXAFS spectra of TiO_2-supported Ru_{100} at Ru K-edge. Reaction conditions: PP_{12}:catalyst = 1:1 mass ratio, 513 K, 20 bar H_2, 4 h.

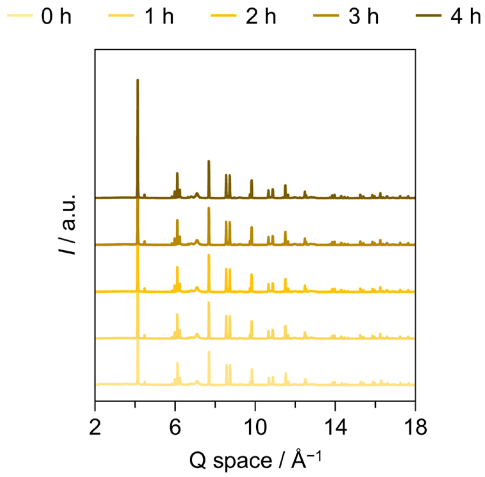

Figure S11. Time-resolved operando HRPD spectra of TiO_2-supported $Co_{10}Ru_{90}$ at full scale. Reaction conditions: PP_{12}:catalyst = 1:1 mass ratio, 513 K, 20 bar H_2, 4 h.

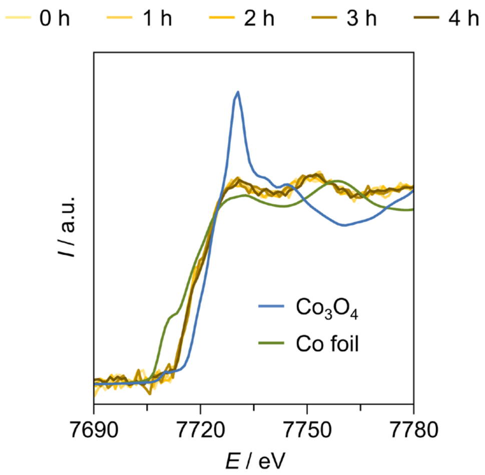

Figure S12. Time-resolved operando XANES spectra of TiO_2-supported $Co_{10}Ru_{90}$ at Co K-edge. Reaction conditions: PP_{12}:catalyst = 1:1 mass ratio, 513 K, 20 bar H_2, 4 h.

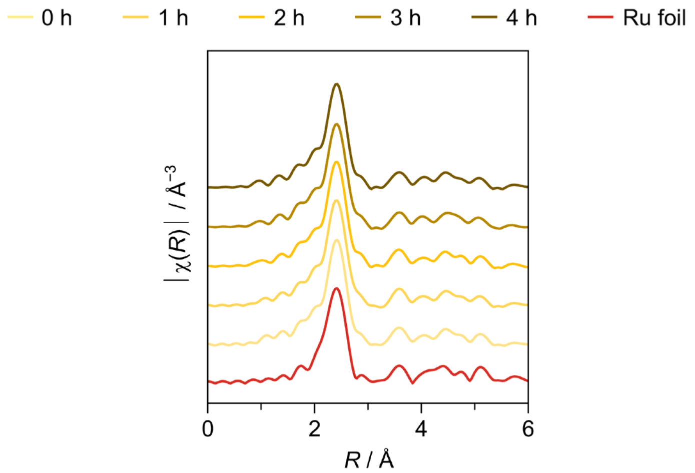

Figure S13. Time-resolved operando EXAFS spectra of TiO_2-supported $Co_{10}Ru_{90}$ at Ru K-edge. Reaction conditions: PP_{12}:catalyst = 1:1 mass ratio, 513 K, 20 bar H_2, 4 h.

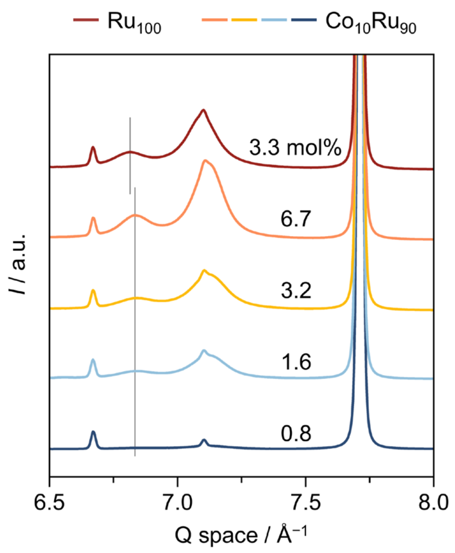

Figure S14. Synchrotron high-resolution powder diffraction, HRPD spectra of Ru_{100} and $Co_{10}Ru_{90}$ catalysts with different total metal contents. The gray lines indicate the characteristic positions of CoRu species.

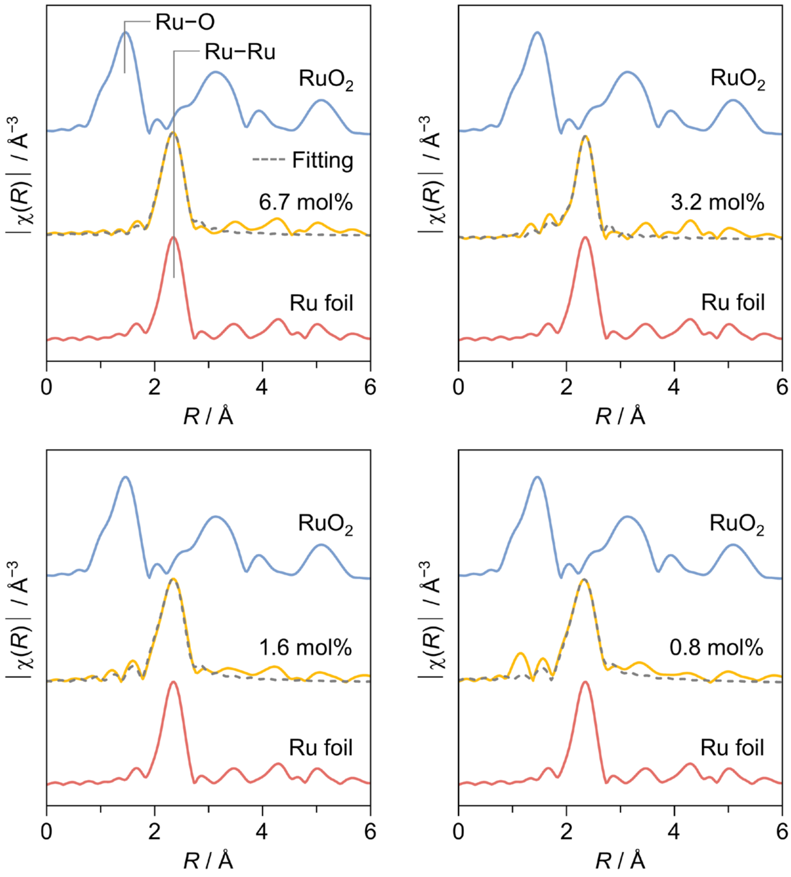

Figure S15. EXAFS profiles and corresponding fittings (dashed lines) for $Co_{10}Ru_{90}$ catalysts with different total metal contents at Ru K-edge. The profiles of standard RuO_2 and Ru foil were included as references.

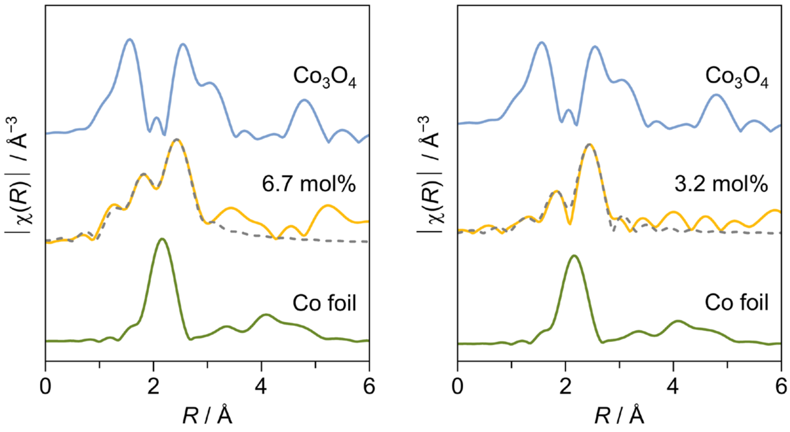

Figure S16. EXAFS profiles and corresponding fittings (dashed lines) of $Co_{10}Ru_{90}$ catalysts with different total metal contents at Co K-edge. The profiles of standard Co_3O_4 and Co foil were included as references.

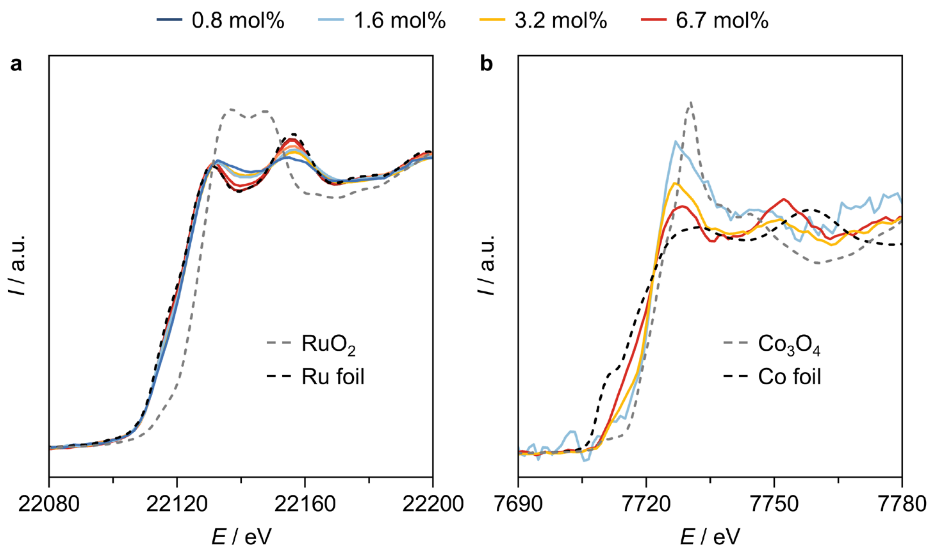

Figure S17. XANES profiles of $Co_{10}Ru_{90}$ catalysts with different total metal contents at (**a**) Ru K-edge, (**b**) Co K-edge. The profiles of standards (dashed lines) including RuO_2, Ru foil, Co_3O_4, and Co foil were included as references.

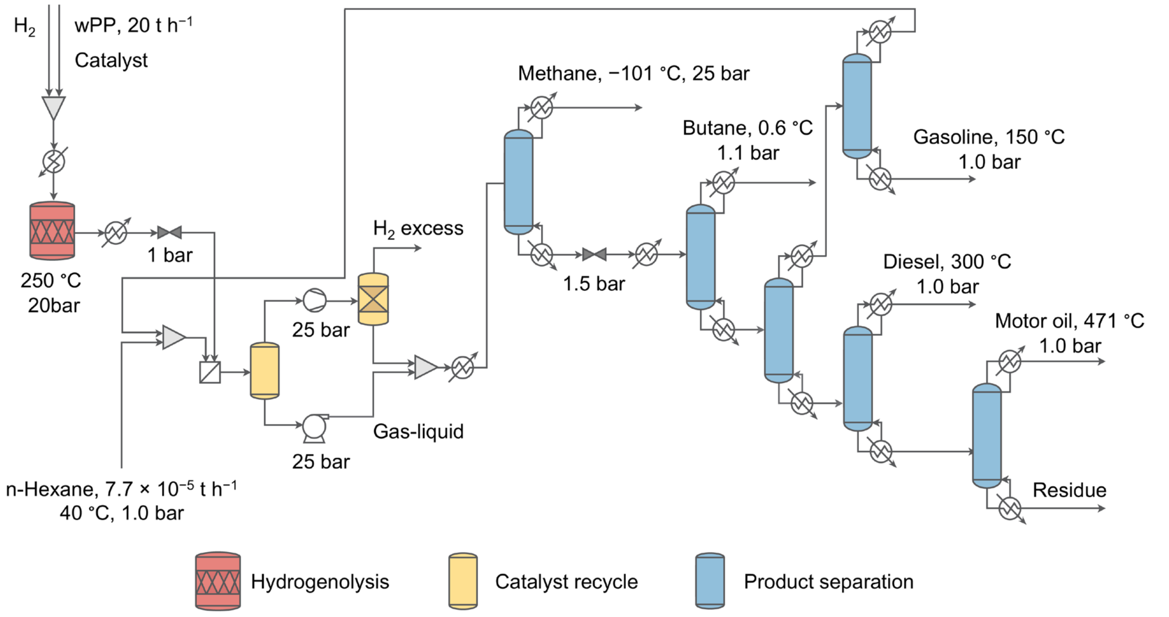

Figure S18. Process flow sheet for recycling 20 t h^{-1} of PP plastics via hydrogenolysis including downstream catalyst recycle and product separation.

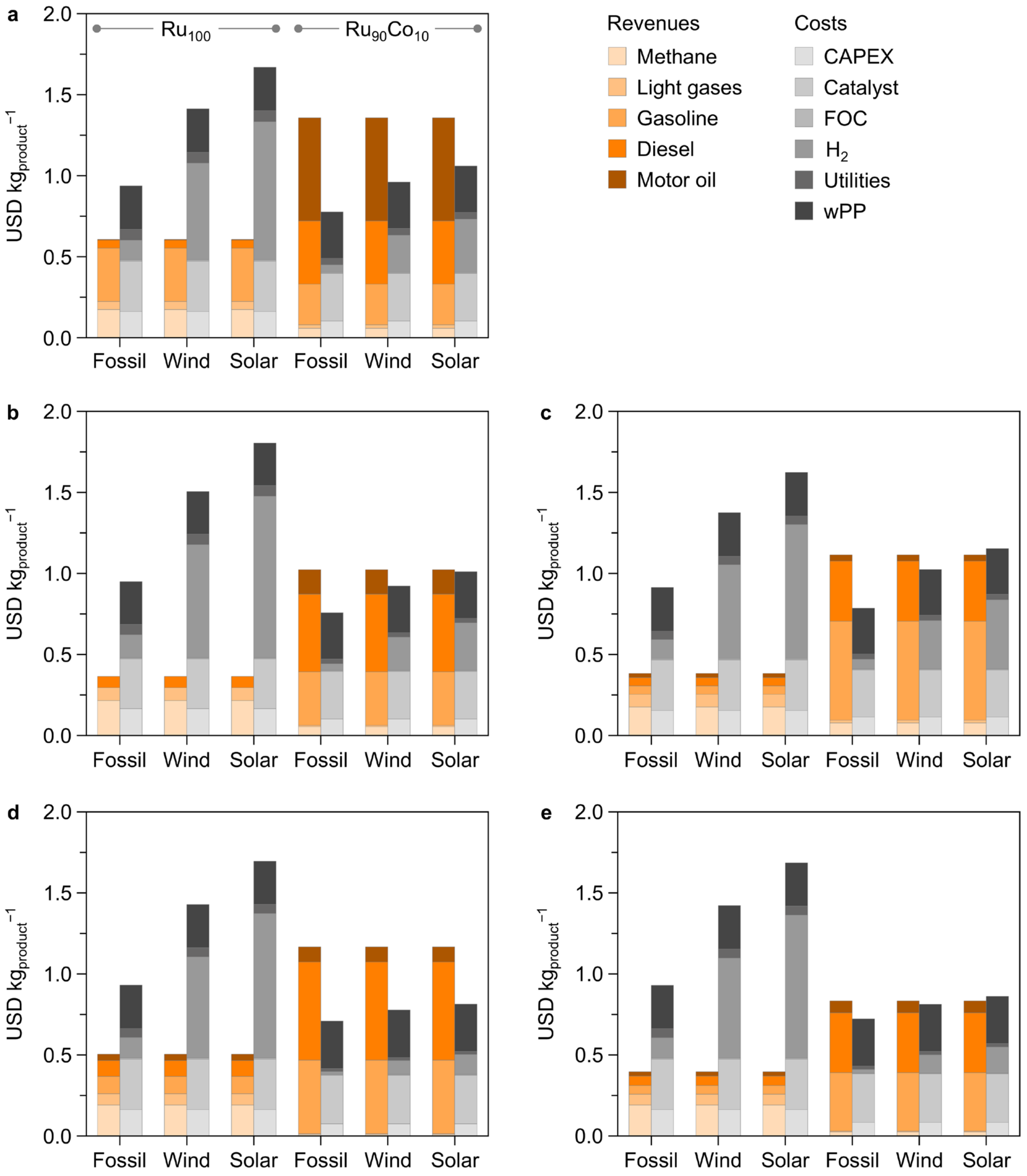

Figure S19. Techno-economic assessment of hydrogenolysis of PP plastics over Ru_{100} and $Co_{10}Ru_{90}$ catalysts. The calculated revenues, costs and profits using different H_2 sources for (**a**) PP_{12}, (**b**) PP_{250}, (**c**) PP bottle, (**d**) shampoo cap and (**e**) yogurt cup. Product yields were obtained from **Table S7** at a reaction time of 12 h for Ru_{100} and 24 h for $Co_{10}Ru_{90}$. Breakdown of revenues and total annualized costs were shown in **Table S21**.

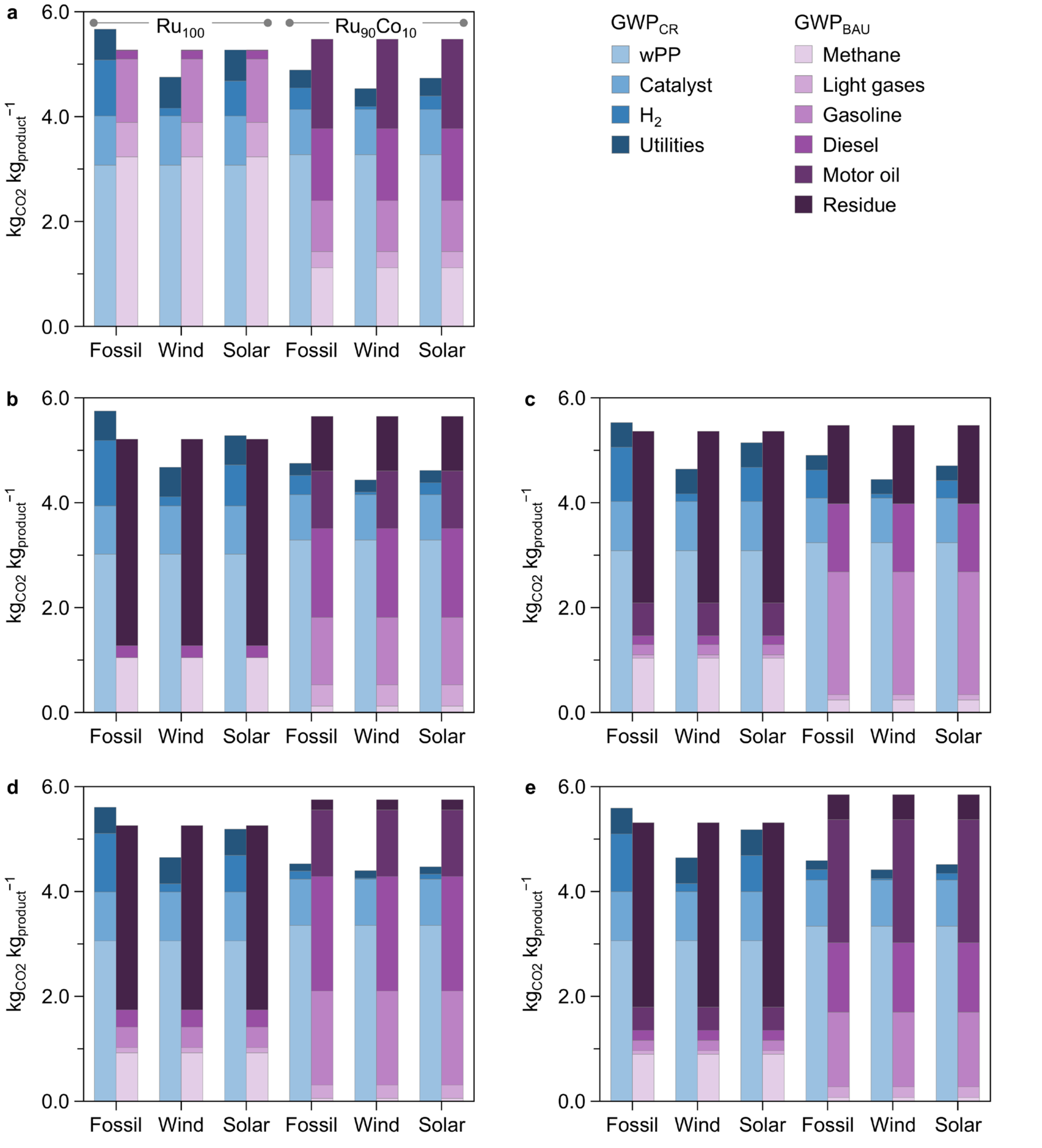

Figure S20. Environmental assessment of large-scale hydrogenolysis of PP plastics over Ru_{100} and $Co_{10}Ru_{90}$ catalysts. The calculated GWP of the hydrogenolysis (chemical recycling) process (GWP_{CR}) and that of the equivalent business-as-usual (BAU) process (GWP_{BAU}) using different H_2 sources for (**a**) PP_{12}, (**b**) PP_{250}, (**c**) PP bottle, (**d**) shampoo cap and (**e**) yogurt cup. CO_2 reductions were calculated as the difference between GWP_{CR} and GWP_{BAU}. Product yields were obtained from **Table S7** at a reaction time of 12 h for Ru_{100} and 24 h for $Co_{10}Ru_{90}$. Contributions to the GWP, CR and GWP, BAU were shown in **Table S22**.

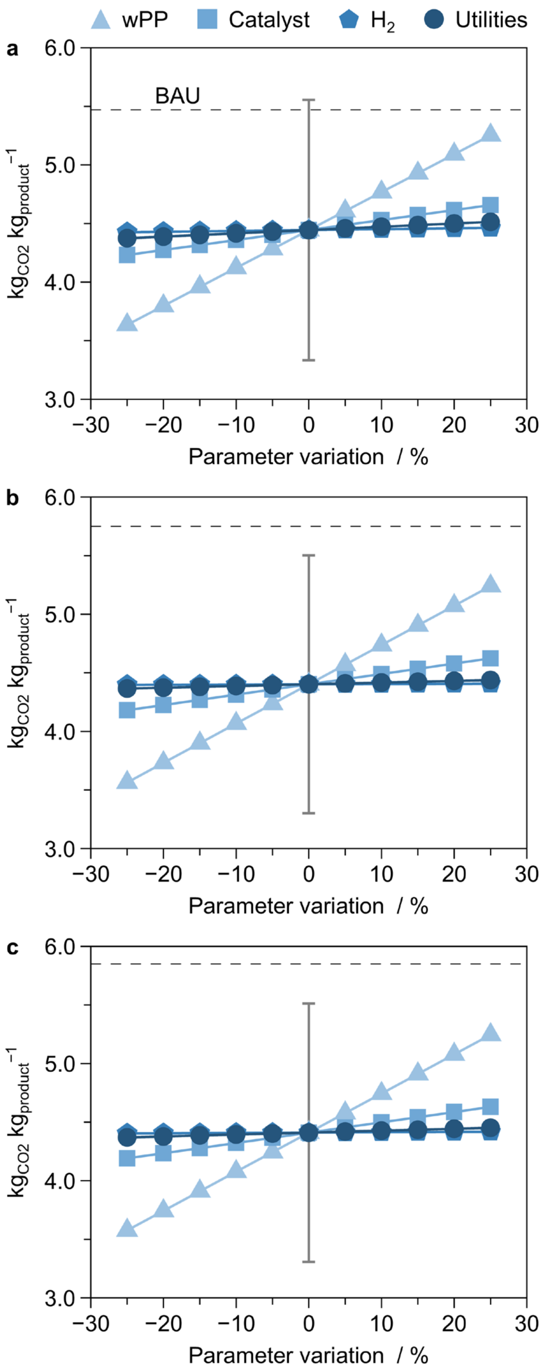

Figure S21. Sensitivity analysis of the GWP of the process described in **Figure S18** for (**a**) PP bottle, (**b**) shampoo cap and (**c**) yogurt cup. The analysis varies the amounts of LCI parameters (i.e., raw materials, electricity, utilities) to assess their effect on the total GWP. Each line of the plot shows the variation of the GWP resulting from changing each LCI parameter by ±25% relative to its nominal value (shown in **Table S23**). The dashed line corresponds to the GWP of the BAU equivalent to the product portfolio generated by the hydrogenolysis process. The maximum and minimum values of the error bar represent the scenarios when all parameters are simultaneously varied by −25% and +25%.

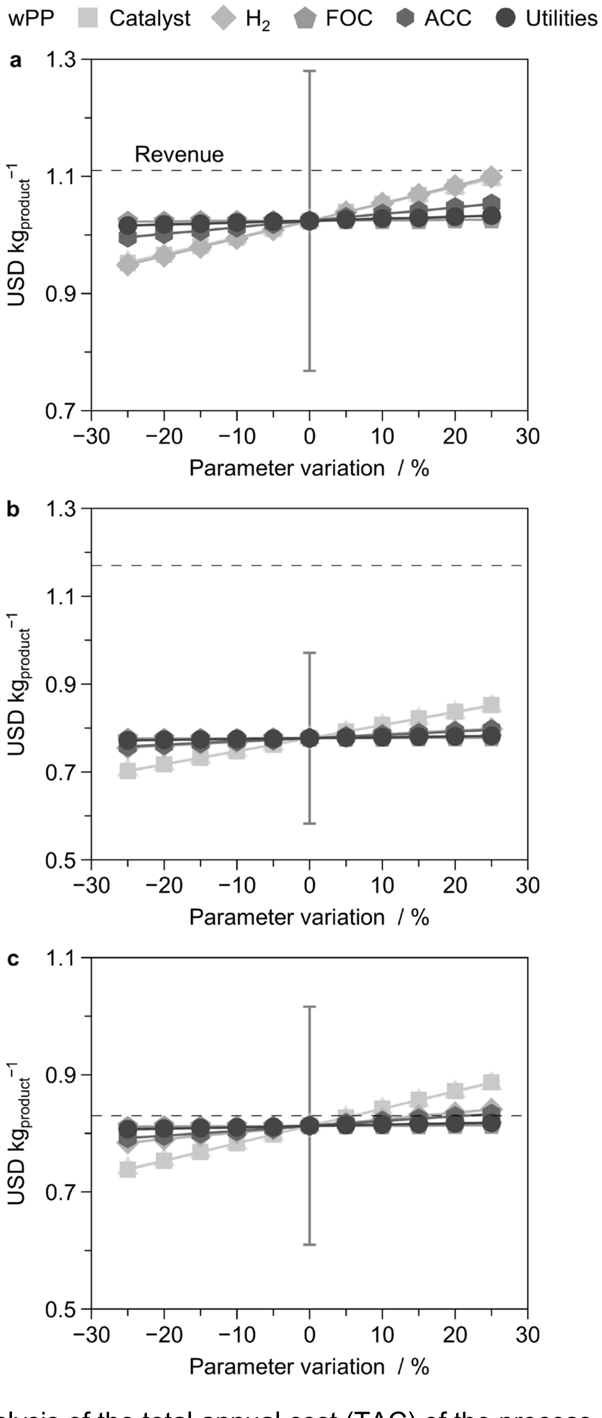

Figure S22. Sensitivity analysis of the total annual cost (TAC) of the process described in **Figure S18** for (**a**) PP bottle, (**b**) shampoo cap and (**c**) yogurt cup. The analysis varies the price of raw materials, electricity, utilities, and other cost parameters to assess their effect on the TAC. Each line of the plot shows the variation of the TAC resulting from the variation of each cost parameter by ±25% relative to its nominal value (shown in **Table S24**). The dashed line corresponds to the market price (revenue) of the product portfolio generated by the hydrogenolysis process and is calculated based on the average market price of each product in. The maximum and minimum values of the error bar represent the scenarios when all parameters are simultaneously varied by −25% and +25%.

Supporting References

[1] A. T. Inc. Physical property methods and models 11.1. technical manual. **2001**.

[2] C. Salah, S. Cobo, J. Pérez-Ramírez, G. Guillén-Gosálbez. Environmental sustainability assessment of hydrogen from waste polymers. *ACS Sustainable Chem. Eng.* **2023**, *11* (8), 3238–3247. DOI: 10.1021/acssuschemeng.2c05729.

[3] R. Sinnott, G. Towler. Costing and project evaluation. *Chem. Eng. Des.* **2020**, 275–369. DOI: 10.1016/b978-0-08-102599-4.00006-0.

[4] Environmental management - life cycle assessment - principles and framework. *International Standards Organization*. **2006**. https://www.iso.org/standard/37456.html.

[5] Environmental management - life cycle assessment - requirements and guidelines. *International Standards Organization*. **2006**. https://www.iso.org/standard/38498.html.

[6] C. Mutel. Brightway: an open source framework for life cycle assessment. *JOSS* **2017**, *2* (12), 236.

[7] G. Wernet, B. Christian, S. Bernhard, et al. The ecoinvent database version 3 (part I): overview and methodology. *Int. J. Life Cycle Assess.* **2016**, *21* (9), 1218–1230.

[8] P. A. Kots, S. Liu, B. C. Vance, et al. Polypropylene plastic waste conversion to lubricants over Ru/TiO_2 Catalysts. *ACS Catal.* **2021**, *11* (13), 8104–8115. DOI: 10.1021/acscatal.1c00874.

[9] S. D. Jaydev, M.-E. Usteri, A. J. Martín, J. Pérez-Ramírez. Identifying selective catalysts in polypropylene hydrogenolysis by decoupling scission pathways. *Chem Catal.* **2023**, *3* (5), 100564. DOI: 10.1016/j.checat.2023.100564.

[10] E. Selvam, Z. O. G. Schyns, J. A. Sun, et al. Conversion of compositionally diverse plastic waste over earth-abundant sulfides. *J. Am. Chem. Soc.* **2025**, *147* (13), 11227–11238. DOI: 10.1021/jacs.4c18001.

[11] J. A. Sun, P. A. Kots, Z. R. Hinton, et al. Size and structure effects of carbon-supported ruthenium nanoparticles on waste polypropylene hydrogenolysis activity, selectivity, and product microstructure. *ACS Catal.* **2024**, *14* (5), 3228–3240. DOI: 10.1021/acscatal.3c05927.

[12] M. Chu, W. Tu, Z. Zhuang, et al. Efficient polyolefin upcycling over single-atom alloy catalyst. *CCS Chem.* **2024**, 1–14. DOI: 10.31635/ccschem.024.202404989.

[13] E. Martelli, T. Kreutz, S. Consonni. Comparison of coal IGCC with and without CO_2 capture and storage: Shell gasification with standard vs. partial water quench. *Energy Procedia* **2009**, *1* (1), 607–614. DOI: 10.1016/j.egypro.2009.01.080.

[14] O. Onel, A. M. Niziolek, C. A. Floudas. Optimal production of light olefins from natural gas via the methanol intermediate. *Ind. Eng. Chem. Res.* **2016**, *55* (11), 3043–3063. DOI: 10.1021/acs.iecr.5b04571.

[15] A. Nabera, I. R. Istrate, A. J. Martín, J. Pérez-Ramírez, G. Guillén-Gosálbez. Energy crisis in Europe enhances the sustainability of green chemicals. *Green Chem.* **2023**, *25* (17), 6603–6611. DOI: 10.1039/d3gc01053h.

[16] Track Isobutane price trend and forecast in top 10 leading countries worldwide. *ChemAnalyst.* **2025**. https://www.chemanalyst.com/Pricing-data/isobutane-1620.

[17] I. Ioannou, J. Javaloyes-Antón, J. A. Caballero, G. Guillén-Gosálbez. Economic and environmental performance of an integrated CO_2 refinery. *ACS Sustainable Chem. Eng.* **2023**, *11* (5), 1949–1961. DOI: 10.1021/acssuschemeng.2c06724.
[18] World Bank commodity price data. *World Bank Group*. **2024**. https://www.worldbank.org/en/research/commodity-markets.
[19] R. Turton, R. C. Bailie, W. B. Whiting, J. A. Shaeiwitz, D. Bhattacharyya. *Analysis, Synthesis, and Design of Chemical Processes*; Pearson Education, **2008**.
[20] Average ruthenium price worldwide from 2013 to 2023. *Statista.com*. **2025**. https://www.statista.com/statistics/1046426/ruthenium-price.
[21] Average cobalt spot price in the United States from 2010 to 2024. *Statista.com*. **2025**. https://www.statista.com/statistics/339743/average-spot-price-of-cobalt-in-the-us.
[22] Price of titanium minerals worldwide from 2018 to 2024, by mineral type. *Statista.com*. **2025**. https://www.statista.com/statistics/1394503/global-price-of-titanium-minerals-by-type.
[23] H. J. Althaus, M. Chudacoff, R. Hischier, et al. *Life cycle inventories of chemicals*; **2007**.
[24] R. Sacchi, T. Terlouw, K. Siala, et al. PRospective EnvironMental Impact asSEment (premise): A streamlined approach to producing databases for prospective life cycle assessment using integrated assessment models. *Renew. Sustain. Energy Rev.* **2022**, *160*, 112311. DOI: 10.1016/j.rser.2022.112311.
